# Supplementary material for: Influence of a Polyherbal Choline Source in Dogs: Body Weight Changes, Blood Metabolites, and Gene Expression
Source: Animals (Basel). 2022 May 20;12(10):1313. doi: 10.3390/ani12101313 (PMC9137459; doi:10.3390/ani12101313)
Supplement: Supplementary file 1 [file animals-12-01313-s001.zip › animals-1655504-supplementary.pdf]

animals

MDPI

Table S1. Differentially Expressed Genes in whole blood of dogs supplemented with polyherbal choline versus choline chloride.

Genbank	Gene Name	Fold
NM_008543	SMAD family member 7(Smad7)	-3.86
NM_007995	ficolin A(Fcna)	-3.56
NM_010249	GA repeat binding protein, beta 1(Gabpb1)	-3.36
U41465	B cell leukemia/lymphoma 6(Bcl6)	-3.34
NM_018760	solute carrier family 4 (anion exchanger), member 4(Slc4a4)	-3.33
NM_009899	chloride channel accessory 3A1(Clca3a1)	-3.27
NM_010602	potassium inwardly rectifying channel, subfamily J, member 11(Kcnj11)	-3.21
AK012039	RIKEN cDNA 2610317O13 gene(2610317O13Rik)	-3.06
AF038500	guanylate cyclase 1, soluble, beta 2(Gucy1b2)	-3.02
AF143539	CD55 molecule, decay accelerating factor for complement(Cd55)	-2.98
AK015139	RIKEN cDNA 4930414N06 gene(4930414N06Rik)	-2.96
AF146224	high mobility group 20B(Hmg20b)	-2.94
AF159856	solute carrier family 38, member 3(Slc38a3)	-2.94
NM_008589	mesoderm posterior 2(Mesp2)	-2.87
AK004665	DEAH (Asp-Glu-Ala-His) box polypeptide 35(Dhx35)	-2.85
X07969	X-linked lymphocyte-regulated(Xlr)	-2.85
NM_011907	three prime repair exonuclease 2(Trex2)	-2.83
K00083	interferon gamma(Ifng)	-2.82
AJ238332	death inducer-obliterator 1(Dido1)	-2.80
NM_010771	matrin 3(Matr3)	-2.79
NM_008095	glioblastoma amplified sequence(Gbas)	-2.79
U22262	apolipoprotein B mRNA editing enzyme, catalytic polypeptide 1(Apobec1)	-2.77
NM_013773	T cell leukemia/lymphoma 1B, 1(Tcl1b1)	-2.77
NM_011915	Wnt inhibitory factor 1(Wif1)	-2.77

NM_011403	solute carrier family 4 (anion exchanger), member 1(Slc4a1)	-2.76
NM_008768	orosomucoid 1(Orm1)	-2.74
NM_009090	polymerase (RNA) II (DNA directed) polypeptide C(Polr2c)	-2.72
AK010304	SET domain containing 6(Setd6)	-2.69
AY027861		-2.69
NM_008517	leukotriene A4 hydrolase(Lta4h)	-2.68
NM_007500	atonal bHLH transcription factor 1(Atoh1)	-2.68
M94307		-2.64
NM_007760	carnitine acetyltransferase(Crat)	-2.64
U58881	protein phosphatase 1, regulatory (inhibitor) subunit 13B(Ppp1r13b)	-2.61
NM_018855	growth arrest specific 8(Gas8)	-2.61
X54327	glutamyl-prolyl-tRNA synthetase(Eprs)	-2.59
X06762	homeobox B7(Hoxb7)	-2.58
NM_019732	runt related transcription factor 3(Runx3)	-2.57
NM_011694	voltage-dependent anion channel 1(Vdac1)	-2.57
NM_007853	delta(4)-desaturase, sphingolipid 1(Degs1)	-2.57
BC013080	grainyhead-like 1 (Drosophila)(Grhl1)	-2.56
NM_010691	ladybird homeobox homolog 1 (Drosophila)(Lbx1)	-2.56
X89594	estrogen related receptor, beta(Esrrb)	-2.56
NM_009175		-2.55
AF214013	Terf1 (TRF1)-interacting nuclear factor 2(Tinf2)	-2.54
NM_008219	hemoglobin Z, beta-like embryonic chain(Hbb-bh1)	-2.53
NM_007951	enhancer of rudimentary homolog (Drosophila)(Erh)	-2.53
NM_013784	phosphatidylinositol glycan anchor biosynthesis, class N(Pign)	-2.53
X07541		-2.53
AK006023	fucose-1-phosphate guanylyltransferase(Fpgt)	-2.53
NM_011304	RuvB-like protein 2(Ruvbl2)	-2.52
NM_021356	growth factor receptor bound protein 2-associated protein 1(Gab1)	-2.52

NM_009195	solute carrier family 12, member 4(Slc12a4)	-2.51
AF140427	T-box 5(Tbx5)	-2.51
AJ131957	SAC3 domain containing 1(Sac3d1)	-2.51
NM_013774	T cell leukemia/lymphoma 1B, 4(Tcl1b4)	-2.50
NM_011609	tumor necrosis factor receptor superfamily, member 1a(Tnfrsf1a)	-2.50
AB041650	FGFR1 oncogene partner 2(Fgfr1op2)	-2.50
NM_008774	poly(A) binding protein, cytoplasmic 1(Pabpc1)	-2.49
U51204	microtubule-associated protein, RP/EB family, member 3(Mapre3)	-2.49
NM_011098	paired-like homeodomain transcription factor 2(Pitx2)	-2.49
X56461	homeobox B6(Hoxb6)	-2.48
NM_019675	stathmin-like 4(Stmn4)	-2.48
AK020669	RIKEN cDNA 9630003H22 gene(9630003H22Rik)	-2.47
AB010321	zinc finger protein 27(Zfp27)	-2.46
NM_008852	paired-like homeodomain transcription factor 3(Pitx3)	-2.45
NM_016717	selenocysteine lyase(Scly)	-2.45
NM_008394	interferon regulatory factor 9(Irf9)	-2.44
NM_008331	interferon-induced protein with tetratricopeptide repeats 1(Ifit1)	-2.43
NM_016804	metaxin 2(Mtx2)	-2.43
AK019796	RIKEN cDNA 4930570D08 gene(4930570D08Rik)	-2.43
NM_030258	G protein-coupled receptor 146(Gpr146)	-2.43
NM_010759	melanoma antigen, family B, 1(Mageb1)	-2.42
AF026565	midline 1(Mid1)	-2.42
NM_008151	G-protein coupled receptor 12(Gpr12)	-2.42
AF172447	afadin, adherens junction formation factor(Afdn)	-2.42
NM_010420	homeobox gene expressed in ES cells(Hesx1)	-2.42
NM_016928	toll-like receptor 5(Tlr5)	-2.41
NM_008644	proline rich, lacrimal 1(Prol1)	-2.41
NM_019494	chemokine (C-X-C motif) ligand 11(Cxcl11)	-2.41

NM_025829	eukaryotic translation initiation factor 4E member 3(Eif4e3)	-2.41
NM_015749	transcobalamin 2(Tcn2)	-2.41
Z12238		-2.41
AB039919	Ras homolog enriched in brain(Rheb)	-2.40
NM_011816	GTPase activating protein (SH3 domain) binding protein 2(G3bp2)	-2.39
NM_008063	solute carrier family 37 (glucose-6-phosphate transporter), member 4(Slc37a4)	-2.39
NM_008718	neuronal PAS domain protein 1(Npas1)	-2.39
NM_008811	pyruvate dehydrogenase E1 alpha 2(Pdha2)	-2.38
NM_009317	T cell acute lymphocytic leukemia 2(Tal2)	-2.38
AK017044	Williams Beuren syndrome chromosome region 25 (human)(Wbscr25)	-2.38
NM_008389	IAP promoted placental gene(Ipp)	-2.37
NM_009896	suppressor of cytokine signaling 1(Socs1)	-2.37
NM_010162	exostoses (multiple) 1(Ext1)	-2.36
NM_009508	solute carrier family 32 (GABA vesicular transporter), member 1(Slc32a1)	-2.36
NM_021363	seminal vesicle secretory protein 3A(Svs3a)	-2.35
AK013664	solute carrier family 17 (sodium-dependent inorganic phosphate cotransporter), member 7(Slc17a7)	-2.35
NM_018827	cytokine receptor-like factor 1(Crlf1)	-2.33
AK016340	glutathione S-transferase, theta 4(Gstt4)	-2.33
NM_008743	nth (endonuclease III)-like 1 (E.coli)(Nthl1)	-2.32
NM_011478	small proline-rich protein 3(Sprr3)	-2.32
NM_021317	DnaJ heat shock protein family (Hsp40) member B7(Dnajb7)	-2.32
NM_011767	zinc finger RNA binding protein(Zfr)	-2.31
AJ231197		-2.31
D13903	protein tyrosine phosphatase, receptor type, D(Ptprd)	-2.31
NM_009246	serine (or cysteine) peptidase inhibitor, clade A, member 1D(Serpina1d)	-2.30
NM_010809	matrix metallopeptidase 3(Mmp3)	-2.30
AF144628	slit homolog 2 (Drosophila)(Slit2)	-2.30
AF121080	solute carrier family 15, member 3(Slc15a3)	-2.30

AK019644	RIKEN cDNA 4930473D10 gene(4930473D10Rik)	-2.30
NM_009460	small ubiquitin-like modifier 1(Sumo1)	-2.29
NM_009852	CD6 antigen(Cd6)	-2.29
AF146593	retinitis pigmentosa 1 (human)(Rp1)	-2.29
AB015423	deltex 2, E3 ubiquitin ligase(Dtx2)	-2.29
NM_010250	gamma-aminobutyric acid (GABA) A receptor, subunit alpha 1(Gabra1)	-2.28
L36434	N-acetylglucosamine-1-phosphate transferase, alpha and beta subunits(Gnptab)	-2.28
NM_008953	BPI fold containing family A, member 2(Bpifa2)	-2.28
NM_009327	HNF1 homeobox A(Hnf1a)	-2.28
NM_011896	sprouty homolog 1 (Drosophila)(Spry1)	-2.28
NM_011930	chloride channel, voltage-sensitive 7(Clcn7)	-2.27
NM_010485	ELAV (embryonic lethal, abnormal vision)-like 1 (Hu antigen R)(Elavl1)	-2.27
AB025408	esterase D/formylglutathione hydrolase(Esd)	-2.27
NM_009528	wingless-type MMTV integration site family, member 7B(Wnt7b)	-2.26
U22056	a disintegrin and metallopeptidase domain 1a(Adam1a)	-2.25
NM_008633	microtubule-associated protein 4(Map4)	-2.25
NM_009044	reticuloendotheliosis oncogene(Rel)	-2.25
NM_007473	aquaporin 7(Aqp7)	-2.25
X99243		-2.25
NM_009949	carnitine palmitoyltransferase 2(Cpt2)	-2.25
NM_021568	poly(rC) binding protein 3(Pcbp3)	-2.25
AV048854	serine peptidase inhibitor, Kazal type 1(Spink1)	-2.25
NM_008814	pancreatic and duodenal homeobox 1(Pdx1)	-2.25
NM_007769	deleted in malignant brain tumors 1(Dmbt1)	-2.25
BF579432		-2.24
NM_008853	praja ring finger 1, E3 ubiquitin protein ligase(Pja1)	-2.24
NM_021430	Rab interacting lysosomal protein-like 1(Rilpl1)	-2.24
NM_010345	growth factor receptor bound protein 10(Grb10)	-2.24

AB049355	family with sequence similarity 129, member A(Fam129a)	-2.24
NM_010812	forkhead box K1(Foxk1)	-2.24
AF020191	DMRT-like family B with proline-rich C-terminal, 1(Dmrtb1)	-2.23
NM_008994	peroxisomal biogenesis factor 2(Pex2)	-2.23
AK005828	RIKEN cDNA 1700010I02 gene(1700010I02Rik)	-2.23
AW489963		-2.22
X83587	REST corepressor 2(Rcor2)	-2.22
NM_021309	SH2 domain containing 2A(Sh2d2a)	-2.22
AJ297743	torsin family 1, member B(Tor1b)	-2.22
AB047758	protease, serine 44(Prss44)	-2.22
AB001423	kinesin family member 16B(Kif16b)	-2.22
NM_019718	ADP-ribosylation factor-like 3(Arl3)	-2.21
NM_009089	polymerase (RNA) II (DNA directed) polypeptide A(Polr2a)	-2.21
NM_021336	small nuclear ribonucleoprotein polypeptide A'(Snrpa1)	-2.21
NM_008799	programmed cell death 2(Pdcd2)	-2.21
AK002854	hydrogen voltage-gated channel 1(Hvcn1)	-2.21
NM_011603	TATA box binding protein-like 1(Tbpl1)	-2.21
NM_009095	ribosomal protein S5(Rps5)	-2.20
NM_019914	myeloid/lymphoid or mixed-lineage leukemia; translocated to, 11(Mllt11)	-2.20
NM_007445	anti-Mullerian hormone(Amh)	-2.20
AK012523	processing of precursor 5, ribonuclease P/MRP family (S. cerevisiae)(Pop5)	-2.19
NM_010361	glutathione S-transferase, theta 2(Gstt2)	-2.19
NM_011487	signal transducer and activator of transcription 4(Stat4)	-2.19
U69176	laminin, alpha 4(Lama4)	-2.19
AF143685	myosin IXb(Myo9b)	-2.18
AA497620		-2.18
NM_016807	syndecan binding protein(Sdcbp)	-2.18
NM_008990	nectin cell adhesion molecule 2(Nectin2)	-2.18

NM_007600	calpain 1(Capn1)	-2.18
AK020866	RIKEN cDNA A930016I07 gene(A930016I07Rik)	-2.17
AK010381	asparaginase like 1(Asrgl1)	-2.17
NM_010184	Fc receptor, IgE, high affinity I, alpha polypeptide(Fcer1a)	-2.17
NM_013743	pyruvate dehydrogenase kinase, isoenzyme 4(Pdk4)	-2.17
NM_019943	poly (A) polymerase beta (testis specific)(Papolb)	-2.17
NM_011798	chemokine (C motif) receptor 1(Xcr1)	-2.16
NM_010445	H6 homeobox 1(Hmx1)	-2.16
NM_013868	heat shock protein family, member 7 (cardiovascular)(Hspb7)	-2.16
NM_009404	tumor necrosis factor (ligand) superfamily, member 9(Tnfsf9)	-2.16
NM_010671	keratin associated protein 13(Krtap13)	-2.16
AJ290946	phosphodiesterase 4D interacting protein (myomegalin)(Pde4dip)	-2.16
NM_010095	early B cell factor 2(Ebf2)	-2.16
U43892	ATP-binding cassette, sub-family B (MDR/TAP), member 7(Abcb7)	-2.16
NM_013604	metaxin 1(Mtx1)	-2.15
NM_008999	RAB23, member RAS oncogene family(Rab23)	-2.15
NM_007453	peroxiredoxin 6(Prdx6)	-2.15
X03766	actin, alpha 1, skeletal muscle(Acta1)	-2.15
M15434	histone cluster 1, H3g(Hist1h3g)	-2.15
NM_021396	programmed cell death 1 ligand 2(Pdcd1lg2)	-2.15
BC005719	RNA binding motif protein 5(Rbm5)	-2.14
NM_011942	lysophospholipase 2(Lypla2)	-2.14
D87968	signal-regulatory protein alpha(Sirpa)	-2.14
AB024336	antigen p97 (melanoma associated) identified by monoclonal antibodies 133.2 and 96.5(Mfi2)	-2.14
NM_008437	napsin A aspartic peptidase(Napsa)	-2.14
NM_009321	tubulin cofactor A(Tbca)	-2.14
NM_007471	amyloid beta (A4) precursor protein(App)	-2.14
AF047716		-2.13

NM_009364	tissue factor pathway inhibitor 2(Tfpi2)	-2.13
NM_008305	perlecan (heparan sulfate proteoglycan 2)(Hspg2)	-2.13
NM_010906	nuclear factor I/X(Nfix)	-2.13
NM_019497	G protein-coupled receptor kinase 4(Grk4)	-2.13
M21836	keratin 8(Krt8)	-2.12
AK005740	protease, serine 51(Prss51)	-2.12
NM_013895	translocase of inner mitochondrial membrane 13(Timm13)	-2.11
NM_008708	N-myristoyltransferase 2(Nmt2)	-2.11
NM_019492	regulator of G-protein signaling 3(Rgs3)	-2.11
X53584	heat shock protein 1 (chaperonin)(Hspd1)	-2.11
NM_010743	interleukin 1 receptor-like 1(Il1rl1)	-2.11
NM_007384	acid-sensing (proton-gated) ion channel 2(Asic2)	-2.10
Z25851		-2.10
U49952	dual-specificity tyrosine-(Y)-phosphorylation regulated kinase 1c(Dyrk1c)	-2.10
NM_010556	interleukin 3(Il3)	-2.09
NM_021876	embryonic ectoderm development(Eed)	-2.09
NM_008409	integral membrane protein 2A(Itm2a)	-2.09
NM_010754	SMAD family member 2(Smad2)	-2.09
NM_010231	flavin containing monooxygenase 1(Fmo1)	-2.09
NM_024245	kinesin family member 23(Kif23)	-2.09
NM_023403	mesoderm development candidate 2(Mesdc2)	-2.09
NM_010745	lymphocyte antigen 86(Ly86)	-2.09
NM_011139	POU domain, class 2, transcription factor 3(Pou2f3)	-2.09
NM_010767	mannan-binding lectin serine peptidase 2(Masp2)	-2.09
BC007161	lysine (K)-specific demethylase 7A(Kdm7a)	-2.09
NM_008587	c-mer proto-oncogene tyrosine kinase(Mertk)	-2.08
NM_008703	neuromedin B receptor(Nmbr)	-2.08
M27583		-2.08

NM_009626	alcohol dehydrogenase 7 (class IV), mu or sigma polypeptide(Adh7)	-2.08
AK005558	placenta expressed transcript 1(Plet1)	-2.08
X73877	phosphorylase kinase alpha 1(Phka1)	-2.08
AK004124	RIKEN cDNA 1110036E04 gene(1110036E04Rik)	-2.07
D87691	eukaryotic translation termination factor 1(Etf1)	-2.07
AF039601	transforming growth factor, beta receptor III(Tgfbr3)	-2.07
NM_009233	SRY (sex determining region Y)-box 1(Sox1)	-2.07
D44443	cDNA sequence BC005685(BC005685)	-2.07
AB001432	kinesin family member 15(Kif15)	-2.06
NM_007524	NK3 homeobox 2(Nkx3-2)	-2.06
NM_007428	angiotensinogen (serpin peptidase inhibitor, clade A, member 8)(Agt)	-2.06
AK007272	RIKEN cDNA 1700124M09 gene(1700124M09Rik)	-2.06
NM_008320	interferon regulatory factor 8(Irf8)	-2.06
NM_011148	protein phosphatase, EF hand calcium-binding domain 2(Ppef2)	-2.06
NM_011529	TRAF family member-associated Nf-kappa B activator(Tank)	-2.06
AF218416	tocopherol (alpha) transfer protein(Ttpa)	-2.06
NM_007553	bone morphogenetic protein 2(Bmp2)	-2.06
NM_011239	RAN binding protein 1(Ranbp1)	-2.05
BC014757	SPRY domain containing 4(Spryd4)	-2.05
NM_011354	small EDRK-rich factor 2(Serf2)	-2.05
AK002310	aph1 homolog C, gamma secretase subunit(Aph1c)	-2.05
NM_021546	N-terminal EF-hand calcium binding protein 3(Necab3)	-2.05
NM_012019	apoptosis-inducing factor, mitochondrion-associated 1(Aifm1)	-2.05
NM_007895	eosinophil-associated, ribonuclease A family, member 2(Ear2)	-2.04
AF317225	Kruppel-like factor 15(Klf15)	-2.04
AK008720	GINS complex subunit 2 (Psf2 homolog)(Gins2)	-2.04
NM_008632	microtubule-associated protein 2(Map2)	-2.04
AF307844	LIM domain and actin binding 1(Lima1)	-2.03

AF219112	pro-platelet basic protein(Ppbp)	-2.03
NM_010259	guanylate binding protein 2b(Gbp2b)	-2.03
AK004440	TAR DNA binding protein(Tardbp)	-2.03
NM_017466	chemokine (C-C motif) receptor-like 2(Ccrl2)	-2.03
NM_007827	CD55 molecule, decay accelerating factor for complement B(Cd55b)	-2.03
AB040490	CXADR-like membrane protein(Clmp)	-2.03
BC013532	chromatin assembly factor 1, subunit B (p60)(Chaf1b)	-2.03
AK019356	RIKEN cDNA 2900079J23 gene(2900079J23Rik)	-2.03
NM_007510	ATPase, H+ transporting, lysosomal V1 subunit E1(Atp6v1e1)	-2.02
NM_009609	actin, gamma, cytoplasmic 1(Actg1)	-2.02
NM_009681	adaptor-related protein complex 3, sigma 1 subunit(Ap3s1)	-2.02
AF023474		-2.02
NM_019868	heterogeneous nuclear ribonucleoprotein H2(Hnrnph2)	-2.02
NM_008906	cathepsin A(Ctsa)	-2.02
AF250145	IZUMO1 receptor, JUNO(Izumo1r)	-2.02
NM_008822	peroxisomal biogenesis factor 7(Pex7)	-2.01
NM_008241	forkhead box G1(Foxg1)	-2.01
NM_011678	ubiquitin specific peptidase 4 (proto-oncogene)(Usp4)	-2.01
NM_011734	sialic acid acetylesterase(Siae)	-2.01
NM_011868	enoyl-Coenzyme A delta isomerase 2(Eci2)	-2.01
NM_010424	hemochromatosis(Hfe)	-2.01
NM_010926	ER membrane protein complex subunit 8(Emc8)	-2.01
NM_009269	serine palmitoyltransferase, long chain base subunit 1(Sptlc1)	-2.01
NM_013610	ninjurin 1(Ninj1)	-2.00
NM_007897	early B cell factor 1(Ebf1)	-2.00
NM_011776	zona pellucida glycoprotein 3(Zp3)	-2.00
NM_011864	3'-phosphoadenosine 5'-phosphosulfate synthase 2(Papss2)	-2.00
NM_020296	RNA binding motif, single stranded interacting protein 1(Rbms1)	-2.00

Y14771	paralemmin(Palm)	-2.00
NM_008843	prolactin induced protein(Pip)	-2.00
AF093559		-2.00
NM_013488	CD4 antigen(Cd4)	-2.00
AF289539	pescadillo ribosomal biogenesis factor 1(Pes1)	-2.00
NM_011260	regenerating islet-derived 3 gamma(Reg3g)	-1.99
AK013418	RIKEN cDNA 2410018L13 gene(2410018L13Rik)	-1.99
NM_008297	heat shock factor 2(Hsf2)	-1.99
AB041599	mitochondrial ribosomal protein L4(Mrpl4)	-1.99
NM_026449	UDP-N-acetyl-alpha-D-galactosamine:polypeptide N-acetylgalactosaminyltransferase-like 5(Galntl5)	-1.99
NM_020492	glycine receptor, alpha 1 subunit(Glra1)	-1.99
NM_010027	D-dopachrome tautomerase(Ddt)	-1.99
NM_009652	thymoma viral proto-oncogene 1(Akt1)	-1.98
NM_009793	calcium/calmodulin-dependent protein kinase IV(Camk4)	-1.98
NM_021479		-1.98
NM_007416	adrenergic receptor, alpha 1b(Adra1b)	-1.98
NM_007692	choline kinase beta(Chkb)	-1.98
NM_020488	gamma-aminobutyric acid (GABA) A receptor, subunit theta(Gabrq)	-1.97
AK015640	N(alpha)-acetyltransferase 30, NatC catalytic subunit(Naa30)	-1.97
NM_010587	intersectin 1 (SH3 domain protein 1A)(Itsn1)	-1.97
NM_009285	stanniocalcin 1(Stc1)	-1.97
AB016197	potassium inwardly-rectifying channel, subfamily J, member 16(Kcnj16)	-1.97
NM_007424	aggrecan(Acan)	-1.97
AK015407	RIKEN cDNA 4930447J18 gene(4930447J18Rik)	-1.97
NM_008117	growth hormone(Gh)	-1.97
NM_011796	calpain 10(Capn10)	-1.97
NM_011909	ubiquitin specific peptidase 18(Usp18)	-1.97
NM_019879	succinate-CoA ligase, GDP-forming, alpha subunit(Suclg1)	-1.97

NM_010441	high mobility group AT-hook 2(Hmga2)	-1.96
AJ409498		-1.96
NM_020267	tripartite motif-containing 44(Trim44)	-1.96
AK015340	complement component 8, beta polypeptide(C8b)	-1.96
AK005964	RIKEN cDNA 1700013M08 gene(1700013M08Rik)	-1.96
AB042027	FERM domain containing 4B(Frmd4b)	-1.96
NM_009078	ribosomal protein L19(Rpl19)	-1.96
NM_010881	nuclear receptor coactivator 1(Ncoa1)	-1.95
AB028071	kidney expressed gene 1(Keg1)	-1.95
NM_011830	inosine 5'-phosphate dehydrogenase 2(Impdh2)	-1.95
NM_013715	COP9 signalosome subunit 5(Cops5)	-1.94
NM_013867	breast cancer anti-estrogen resistance 3(Bcar3)	-1.94
Z12176		-1.94
AK021260	RNA binding motif protein 12 B2(Rbm12b2)	-1.94
NM_007982	PTK2 protein tyrosine kinase 2(Ptk2)	-1.94
NM_007492	aristaless related homeobox(Arx)	-1.94
NM_019709	membrane-bound transcription factor peptidase, site 1(Mbtps1)	-1.94
NM_018811	abhydrolase domain containing 2(Abhd2)	-1.94
NM_013777	aldo-keto reductase family 1, member C12(Akr1c12)	-1.94
NM_007989	forkhead box H1(Foxh1)	-1.94
NM_011550	MAX-like protein X(Mlx)	-1.94
NM_013499	complement component (3b/4b) receptor 1-like(Cr1l)	-1.93
NM_009093	ribosomal protein S29(Rps29)	-1.93
NM_011880	regulator of G protein signaling 7(Rgs7)	-1.93
U11075	potassium inwardly-rectifying channel, subfamily J, member 4(Kcnj4)	-1.93
NM_015780	complement factor H-related 1(Cfhr1)	-1.93
NM_011224	muscle glycogen phosphorylase(Pygm)	-1.93
AF213393	ATP-binding cassette, sub-family A (ABC1), member 8b(Abca8b)	-1.92

AF057367	ubiquitously transcribed tetratricopeptide repeat gene, Y chromosome(Uty)	-1.92
NM_009819	catenin (cadherin associated protein), alpha 2(Ctnna2)	-1.92
AK003710	RIKEN cDNA 1110014L15 gene(1110014L15Rik)	-1.92
NM_010740	CD93 antigen(Cd93)	-1.92
NM_008986	polymerase I and transcript release factor(Ptrf)	-1.92
NM_009079	ribosomal protein L22(Rpl22)	-1.92
AF221099	carcinoembryonic antigen-related cell adhesion molecule 11(Ceacam11)	-1.92
NM_009197	solute carrier family 16 (monocarboxylic acid transporters), member 2(Slc16a2)	-1.91
AK015991	RIKEN cDNA 4930538K18 gene(4930538K18Rik)	-1.91
AJ250189	ring finger protein 31(Rnf31)	-1.91
NM_009575	zinc finger protein of the cerebellum 3(Zic3)	-1.91
NM_017382	RAB11A, member RAS oncogene family(Rab11a)	-1.91
M55181	preproenkephalin(Penk)	-1.91
NM_008026	Friend leukemia integration 1(Fli1)	-1.91
NM_008820	peptidase D(Pepd)	-1.91
NM_010430	hypermethylated in cancer 1(Hic1)	-1.91
X75927	ATP-binding cassette, sub-family A (ABC1), member 2(Abca2)	-1.90
AF296075	intraflagellar transport 122(Ift122)	-1.90
NM_011317	KH domain containing, RNA binding, signal transduction associated 1(Khdrbs1)	-1.90
NM_021477	RNA binding protein, fox-1 homolog (C. elegans) 1(Rbfox1)	-1.90
NM_008128	gap junction protein, beta 6(Gjb6)	-1.90
NM_013457	adducin 1 (alpha)(Add1)	-1.90
AB041554	sperm associated antigen 4(Spag4)	-1.90
NM_009822	runt-related transcription factor 1; translocated to, 1 (cyclin D-related)(Runx1t1)	-1.90
NM_016981	solute carrier family 9 (sodium/hydrogen exchanger), member 1(Slc9a1)	-1.90
NM_013756	defensin beta 3(Defb3)	-1.90
NM_016791	nuclear factor of activated T cells, cytoplasmic, calcineurin dependent 1(Nfatc1)	-1.90
NM_019404	arginine vasopressin receptor 2(Avpr2)	-1.90

NM_017475	Ras-related GTP binding C(Rragc)	-1.90
AF058955	succinate-Coenzyme A ligase, ADP-forming, beta subunit(Sucla2)	-1.89
AF266505	pseudouridine synthase 3(Pus3)	-1.89
AK006064	RIKEN cDNA 1700017G19 gene(1700017G19Rik)	-1.89
NM_013680	synapsin I(Syn1)	-1.89
AK014086	golgi integral membrane protein 4(Golim4)	-1.89
AK014047	lipase, endothelial(Lipg)	-1.89
NM_021289	submaxillary gland androgen regulated protein 2(Smr2)	-1.89
AK010153	titin(Ttn)	-1.89
D13905	protein tyrosine phosphatase, receptor type, D(Ptprd)	-1.89
BC002235	trafficking protein particle complex 12(Trappc12)	-1.89
NM_026668	leucine-rich repeats and IQ motif containing 4(Lrriq4)	-1.88
NM_020574	potassium voltage-gated channel, Isk-related subfamily, gene 3(Kcne3)	-1.88
AK009333	SH3 and PX domains 2A(Sh3pxd2a)	-1.88
NM_010276	GTP binding protein (gene overexpressed in skeletal muscle)(Gem)	-1.88
NM_021442	MDS1 and EVI1 complex locus(Mecom)	-1.88
NM_013699	upstream binding protein 1(Ubp1)	-1.88
NM_011397	solute carrier family 23 (nucleobase transporters), member 1(Slc23a1)	-1.88
AK013364	protection of telomeres 1B(Pot1b)	-1.88
NM_009126	serine (or cysteine) peptidase inhibitor, clade B (ovalbumin), member 3A(Serpinb3a)	-1.88
U25109		-1.88
NM_008340	insulin-like growth factor binding protein, acid labile subunit(Igfals)	-1.88
NM_019403	ring finger protein 5(Rnf5)	-1.88
NM_018813	cleavage and polyadenylation specificity factor 3(Cpsf3)	-1.88
AK014259	osteoglycin(Ogn)	-1.87
AK019586	RIKEN cDNA 4930428D20 gene(4930428D20Rik)	-1.87
U07207		-1.87
NM_009787	protein disulfide isomerase associated 4(Pdia4)	-1.87

NM_011185	proteasome (prosome, macropain) subunit, beta type 1(Psmb1)	-1.87
NM_012035	transient receptor potential cation channel, subfamily C, member 7(Trpc7)	-1.87
NM_007976	coagulation factor V(F5)	-1.87
NM_009997	cytochrome P450, family 2, subfamily a, polypeptide 4(Cyp2a4)	-1.87
NM_010247	X-ray repair complementing defective repair in Chinese hamster cells 6(Xrcc6)	-1.87
AK017992	Wolf-Hirschhorn syndrome candidate 1 (human)(Whsc1)	-1.87
NM_009281	zinc finger protein 143(Zfp143)	-1.87
AK007294	transformation related protein 53 target 5(Trp53tg5)	-1.87
BC003331	cDNA sequence BC003331(BC003331)	-1.86
AK008571	RIKEN cDNA 2010315B03 gene(2010315B03Rik)	-1.86
L21707	receptor-like tyrosine kinase(Ryk)	-1.86
D26157	prostaglandin I receptor (IP)(Ptgir)	-1.86
NM_008011	fibroblast growth factor receptor 4(Fgfr4)	-1.86
NM_011858	teneurin transmembrane protein 4(Tenm4)	-1.86
NM_007718	chemokine (C-C motif) receptor 1-like 1(Ccr1l1)	-1.86
M94293	ubiquitin specific peptidase 7(Usp7)	-1.86
AK019544	maestro heat-like repeat family member 9(Mroh9)	-1.86
U55417		-1.86
NM_013864	N-myc downstream regulated gene 2(Ndrg2)	-1.86
NM_026010	lipoyl(octanoyl) transferase 2 (putative)(Lipt2)	-1.86
AF041060	GrpE-like 2, mitochondrial(Grpel2)	-1.85
Z12503		-1.85
U57554	melatonin receptor 1B(Mtnr1b)	-1.85
NM_013459	complement factor D (adipsin)(Cfd)	-1.85
AF041409	integrin alpha 8(Itga8)	-1.85
NM_008900	POU domain, class 3, transcription factor 3(Pou3f3)	-1.85
BC004683	zinc finger, DHHC domain containing 8(Zdhhc8)	-1.85
NM_019660	MYC binding protein(Mycbp)	-1.85

AJ249901	SPARC related modular calcium binding 2(Smoc2)	-1.85
NM_007770	cone-rod homeobox(Crx)	-1.85
NM_013746	pleckstrin homology domain containing, family B (evectins) member 1(Plekhb1)	-1.84
NM_013930	aminoadipate-semialdehyde synthase(Aass)	-1.84
S74391	PTK6 protein tyrosine kinase 6(Ptk6)	-1.84
AJ278461	matrix metallopeptidase 1b (interstitial collagenase)(Mmp1b)	-1.84
NM_007683	centromere protein C1(Cenpc1)	-1.84
NM_008205	histocompatibility 2, M region locus 9(H2-M9)	-1.84
NM_007591	calreticulin(Calr)	-1.84
NM_009532	X-ray repair complementing defective repair in Chinese hamster cells 1(Xrcc1)	-1.84
AF353245	unconventional SNARE in the ER 1 homolog (S. cerevisiae)(Use1)	-1.84
NM_011377	single-minded homolog 2 (Drosophila)(Sim2)	-1.84
S72511		-1.84
NM_025575	SYS1 Golgi-localized integral membrane protein homolog (S. cerevisiae)(Sys1)	-1.84
NM_011859	odd-skipped related 1 (Drosophila)(Osr1)	-1.83
NM_016763	hydroxysteroid (17-beta) dehydrogenase 10(Hsd17b10)	-1.83
AF093699		-1.83
AF271360	serine/threonine kinase 4(Stk4)	-1.83
AK016871	RIKEN cDNA 4933422E07 gene(4933422E07Rik)	-1.83
NM_011026	purinergic receptor P2X, ligand-gated ion channel 4(P2rx4)	-1.83
NM_011474	small proline-rich protein 2H(Sprr2h)	-1.83
X92592	forkhead box B1(Foxb1)	-1.83
AF214575	glycine receptor, alpha 3 subunit(Glra3)	-1.83
AK014502	RAN binding protein 10(Ranbp10)	-1.83
AF226324	solute carrier organic anion transporter family, member 3a1(Slco3a1)	-1.83
NM_011959	origin recognition complex, subunit 5(Orc5)	-1.83
AB010373	zinc finger protein 975(Zfp975)	-1.83
AK006578	RIKEN cDNA 1700031F05 gene(1700031F05Rik)	-1.83

AK013157	F-box protein 21(Fbxo21)	-1.82
NM_008070	gamma-aminobutyric acid (GABA) A receptor, subunit beta 2(Gabrb2)	-1.82
NM_026345	MANSC domain containing 1(Mansc1)	-1.82
NM_018740	elongator acetyltransferase complex subunit 5(Elp5)	-1.82
NM_018770	cell adhesion molecule 1(Cadm1)	-1.82
AF291494		-1.82
NM_023233	tripartite motif-containing 13(Trim13)	-1.82
AK016211	RIKEN cDNA 4930563N14 gene(4930563N14Rik)	-1.82
NM_008243	macrophage stimulating 1 (hepatocyte growth factor-like)(Mst1)	-1.82
NM_013492	clusterin(Clu)	-1.82
NM_015810	polymerase (DNA directed), gamma 2, accessory subunit(Polg2)	-1.82
AF278712	allantoicase(Allc)	-1.82
NM_019447	hepatocyte growth factor activator(Hgfac)	-1.82
NM_008380	inhibin beta-A(Inhba)	-1.82
AK018406	RIKEN cDNA 8430413B15 gene(8430413B15Rik)	-1.82
NM_016861	PDZ and LIM domain 1 (elfin)(Pdlim1)	-1.82
AF176528	F-box and WD-40 domain protein 14(Fbxw14)	-1.82
X62705		-1.81
NM_010419	hairy and enhancer of split 5 (Drosophila)(Hes5)	-1.81
NM_022030	synaptic vesicle glycoprotein 2 a(Sv2a)	-1.81
NM_025768	GH regulated TBC protein 1(Grtp1)	-1.81
NM_009371	transforming growth factor, beta receptor II(Tgfbr2)	-1.81
NM_009011	RAD23 homolog B, nucleotide excision repair protein(Rad23b)	-1.81
BC003350	G protein pathway suppressor 1(Gps1)	-1.81
U08819	microtubule-associated protein 4(Map4)	-1.81
NM_011261	reelin(Reln)	-1.81
NM_030253	poly (ADP-ribose) polymerase family, member 9(Parp9)	-1.81
AJ271055	Iroquois related homeobox 6 (Drosophila)(Irx6)	-1.81

NM_008051	fucosyltransferase 1(Fut1)	-1.81
AB010374	zinc finger protein 738(Zfp738)	-1.81
AB018421	cytochrome P450, family 4, subfamily a, polypeptide 10(Cyp4a10)	-1.81
AK007951	trafficking protein particle complex 2(Trappc2)	-1.80
AF089721	smoothened, frizzled class receptor(Smo)	-1.80
NM_009623	adenylate cyclase 8(Adcy8)	-1.80
NM_011549	transcription factor EB(Tfeb)	-1.80
NM_009548	ring finger protein 112(Rnf112)	-1.80
NM_008426	potassium inwardly-rectifying channel, subfamily J, member 3(Kcnj3)	-1.80
NM_011812	fibulin 5(Fbln5)	-1.80
NM_007559	bone morphogenetic protein 8b(Bmp8b)	-1.80
AK021388	RIKEN cDNA E130114A11 gene(E130114A11Rik)	-1.80
NM_015732	axin 2(Axin2)	-1.79
L26238	labial homeobox homolog (Drosophila)(Labx)	-1.79
NM_010012	cytochrome P450, family 8, subfamily b, polypeptide 1(Cyp8b1)	-1.79
NM_009409	topoisomerase (DNA) II beta(Top2b)	-1.79
NM_015820	heparan sulfate 6-O-sulfotransferase 3(Hs6st3)	-1.79
NM_009771	beta-transducin repeat containing protein(Btrc)	-1.79
NM_010629	kinesin-associated protein 3(Kifap3)	-1.79
AK005303	anaphase promoting complex subunit 10(Anapc10)	-1.79
NM_012025	Rac GTPase-activating protein 1(Racgap1)	-1.79
NM_023644	methylcrotonoyl-Coenzyme A carboxylase 1 (alpha)(Mccc1)	-1.79
D13904	protein tyrosine phosphatase, receptor type, D(Ptprd)	-1.79
NM_007916	DEAD (Asp-Glu-Ala-Asp) box polypeptide 19a(Ddx19a)	-1.79
AF090373	gamma-aminobutyric acid (GABA) A receptor, subunit alpha 4(Gabra4)	-1.79
NM_008977	protein tyrosine phosphatase, non-receptor type 2(Ptpn2)	-1.79
AJ275988	trans-acting transcription factor 6(Sp6)	-1.79
AK006870	RIKEN cDNA 1700063H06 gene(1700063H06Rik)	-1.79

NM_018734	guanylate binding protein 3(Gbp3)	-1.79
NM_011346	selectin, lymphocyte(Sell)	-1.78
NM_009810	caspase 3(Casp3)	-1.78
BC003957	family with sequence similarity 131, member A(Fam131a)	-1.78
NM_007719	chemokine (C-C motif) receptor 7(Ccr7)	-1.78
NM_008188	THUMP domain containing 3(Thumpd3)	-1.78
AK014828	tetratricopeptide repeat domain 6(Ttc6)	-1.78
AK007145	family with sequence similarity 65, member B(Fam65b)	-1.78
NM_008548	mannosidase 1, alpha(Man1a)	-1.78
NM_007390	cholinergic receptor, nicotinic, alpha polypeptide 7(Chrna7)	-1.78
AK018520	Rho GTPase activating protein 42(Arhgap42)	-1.78
NM_011791	ash2 (absent, small, or homeotic)-like (Drosophila)(Ash2l)	-1.78
NM_011405	solute carrier family 7 (cationic amino acid transporter, y+ system), member 7(Slc7a7)	-1.78
NM_008600	major intrinsic protein of lens fiber(Mip)	-1.78
AF045562	ATP-binding cassette, sub-family B (MDR/TAP), member 6(Abcb6)	-1.77
AK016767	RIKEN cDNA 4933411E02 gene(4933411E02Rik)	-1.77
AK020271	RIKEN cDNA 9130201A16 gene(9130201A16Rik)	-1.77
NM_010176	fumarylacetoacetate hydrolase(Fah)	-1.77
NM_018774	polyhomeotic-like 2 (Drosophila)(Phc2)	-1.77
Z78162		-1.77
NM_021281	cathepsin S(Ctss)	-1.77
BC011413	40S ribosomal protein S20 pseudogene(Gm6607)	-1.77
NM_010336	lysophosphatidic acid receptor 1(Lpar1)	-1.77
X56574	retinoic acid receptor, beta(Rarb)	-1.77
M93663		-1.77
NM_019687	solute carrier family 22 (organic cation transporter), member 4(Slc22a4)	-1.76
U92454	WW domain binding protein 5(Wbp5)	-1.76
AF270495	adenosine deaminase, RNA-specific, B2(Adarb2)	-1.76

AK019795	PRAME family member 12(Pramef12)	-1.76
NM_010516	cysteine rich protein 61(Cyr61)	-1.76
AK019493		-1.76
NM_007513	solute carrier family 7 (cationic amino acid transporter, y+ system), member 1(Slc7a1)	-1.76
NM_010219	FK506 binding protein 4(Fkbp4)	-1.76
U01139	LTV1 ribosome biogenesis factor(Ltv1)	-1.76
AB010361	zinc finger protein 160(Zfp160)	-1.75
X16670		-1.75
AB036341		-1.75
AK006388	family with sequence similarity 217, member A(Fam217a)	-1.75
AK021288	S phase cyclin A-associated protein in the ER(Scaper)	-1.75
NM_010488	ELAV (embryonic lethal, abnormal vision, Drosophila)-like 4 (Hu antigen D)(Elavl4)	-1.75
NM_010847	MAX interactor 1, dimerization protein(Mxi1)	-1.75
NM_011116	phospholipase D family, member 3(Pld3)	-1.75
NM_008615	malic enzyme 1, NADP(+)-dependent, cytosolic(Me1)	-1.75
M97864		-1.75
M36880		-1.75
NM_019822	adhesion regulating molecule 1(Adrm1)	-1.75
M28513	zinc finger protein 58(Zfp58)	-1.75
NM_009678	adaptor protein complex AP-1, mu 2 subunit(Ap1m2)	-1.75
L17069	lysine (K)-specific methyltransferase 2A(Kmt2a)	-1.75
X75536		-1.75
AK007799		-1.74
NM_021331	glucose-6-phosphatase, catalytic, 2(G6pc2)	-1.74
AF112300	LEM domain containing 3(Lemd3)	-1.74
NM_008664	myomesin 2(Myom2)	-1.74
NM_018874	pancreatic lipase related protein 1(Pnliprp1)	-1.74
NM_008288	hydroxysteroid 11-beta dehydrogenase 1(Hsd11b1)	-1.74

NM_008825	6-phosphofructo-2-kinase/fructose-2,6-biphosphatase 2(Pfkfb2)	-1.74
AF036898	polymerase (DNA directed), epsilon 2 (p59 subunit)(Pole2)	-1.74
NM_007462	adenomatosis polyposis coli(Apc)	-1.74
NM_010937	neuroblastoma ras oncogene(Nras)	-1.74
NM_010024	dopachrome tautomerase(Dct)	-1.74
NM_007846	defensin, alpha, related sequence 12(Defa-rs12)	-1.74
AF242319	centrosomal protein 70(Cep70)	-1.73
NM_009134	sodium channel, voltage-gated, type X, alpha(Scn10a)	-1.73
M91458	sterol carrier protein 2, liver(Scp2)	-1.73
X62895		-1.73
NM_007927	emerin(Emd)	-1.73
NM_007534	B cell leukemia/lymphoma 2 related protein A1b(Bcl2a1b)	-1.73
AF263743	Erbb2 interacting protein(Erbin)	-1.73
AJ250689		-1.73
NM_009557	zinc finger protein 46(Zfp46)	-1.73
AF118273	cDNA sequence BC018473(BC018473)	-1.73
X02676		-1.73
AF093261	homer scaffolding protein 3(Homer3)	-1.73
AL355704	high mobility group 20A(Hmg20a)	-1.73
NM_020522		-1.73
NM_010149	erythropoietin receptor(Epor)	-1.73
X89693	olfactory receptor 242(Olfr242)	-1.73
AF373410	CD209c antigen(Cd209c)	-1.73
AK003894	glycosyltransferase 8 domain containing 2(Glt8d2)	-1.73
AK020088	RIKEN cDNA 6030498E09 gene(6030498E09Rik)	-1.72
AK013656	RIKEN cDNA 2900046H12 gene(2900046H12Rik)	-1.72
AF158744	prolactin family8, subfamily a, member 9(Prl8a9)	-1.72
NM_009278	Sjogren syndrome antigen B(Ssb)	-1.72

NM_009036	recombination signal binding protein for immunoglobulin kappa J region-like(Rbpjl)	-1.72
NM_008235	hairy and enhancer of split 1 (Drosophila)(Hes1)	-1.72
NM_008653	myosin binding protein C, cardiac(Mybpc3)	-1.72
AB035383	ADP-ribosylation factor-like 6 interacting protein 4(Arl6ip4)	-1.72
NM_018862	1-acylglycerol-3-phosphate O-acyltransferase 1 (lysophosphatidic acid acyltransferase, alpha)(Agpat1)	-1.72
AF290473	nudE neurodevelopment protein 1(Nde1)	-1.72
NM_009163	sphingosine phosphate lyase 1(Sgpl1)	-1.72
NM_013881	unc-51 like kinase 2(Ulk2)	-1.72
NM_016909	translin-associated factor X(Tsnax)	-1.72
AK007084	RIKEN cDNA 1700095J12 gene(1700095J12Rik)	-1.72
NM_011978	solute carrier family 27 (fatty acid transporter), member 2(Slc27a2)	-1.72
NM_019791	melanoma antigen, family D, 1(Maged1)	-1.72
NM_011935	estrogen-related receptor gamma(Esrrg)	-1.72
BC004586		-1.72
NM_008599	chemokine (C-X-C motif) ligand 9(Cxcl9)	-1.72
AK013968	RIKEN cDNA 3110001N23 gene(3110001N23Rik)	-1.72
AF154337	G protein-coupled receptor 137B, pseudogene(Gpr137b-ps)	-1.71
AJ276690	B-box and SPRY domain containing(Bspry)	-1.71
M12052	CD8 antigen, alpha chain(Cd8a)	-1.71
NM_009198	solute carrier family 17 (sodium phosphate), member 1(Slc17a1)	-1.71
AJ293727	kyphoscoliosis peptidase(Ky)	-1.71
NM_016882	squamous cell carcinoma antigen recognized by T cells 1(Sart1)	-1.71
NM_008678	nuclear receptor coactivator 2(Ncoa2)	-1.71
Y11356	apolipoprotein H(Apoh)	-1.71
M55697		-1.71
AF237669	ral guanine nucleotide dissociation stimulator-like 3(Rgl3)	-1.71
NM_007446	amylase 1, salivary(Amy1)	-1.71
NM_008408	STT3, subunit of the oligosaccharyltransferase complex, homolog A (S. cerevisiae)(Stt3a)	-1.70

NM_009099	tripartite motif-containing 30A(Trim30a)	-1.70
NM_011835	katanin p60 (ATPase-containing) subunit A1(Katna1)	-1.70
AK015442	RIKEN cDNA 4930451G09 gene(4930451G09Rik)	-1.70
AJ249492	interleukin 10-related T cell-derived inducible factor beta(Iltifb)	-1.70
NM_021543	protocadherin 8(Pcdh8)	-1.70
NM_011596	ATPase, H+ transporting, lysosomal V0 subunit A2(Atp6v0a2)	-1.70
Y17851	ganglioside-induced differentiation-associated-protein 2(Gdap2)	-1.70
NM_007905	polyhomeotic-like 1 (Drosophila)(Phc1)	-1.70
AF282280		-1.70
NM_011717	widely-interspaced zinc finger motifs(Wiz)	-1.70
D45212	protein tyrosine phosphatase, receptor type, J(Ptprj)	-1.70
AJ010223	dystrobrevin, beta(Dtnb)	-1.70
NM_008264	homeobox A13(Hoxa13)	-1.70
NM_019697	potassium voltage-gated channel, Shal-related family, member 2(Kcnd2)	-1.70
AB010369	zinc finger protein 868(Zfp868)	-1.70
M30432		-1.70
AB010370	predicted gene, 17449(Gm17449)	-1.69
NM_010780	chymase 1, mast cell(Cma1)	-1.69
NM_013790	ATP-binding cassette, sub-family C (CFTR/MRP), member 5(Abcc5)	-1.69
AF399754	NADPH oxidase organizer 1(Noxo1)	-1.69
NM_013730	signaling lymphocytic activation molecule family member 1(Slamf1)	-1.69
M94299		-1.69
NM_016771	sulfotransferase family 1D, member 1(Sult1d1)	-1.69
AF022804	phospholipase C, beta 4(Plcb4)	-1.69
NM_009727	ATPase, aminophospholipid transporter (APLT), class I, type 8A, member 1(Atp8a1)	-1.69
AK016170	thyroid hormone receptor interactor 4(Trip4)	-1.69
NM_007794		-1.69
X63927		-1.69

NM_013731	serum/glucocorticoid regulated kinase 2(Sgk2)	-1.69
AK004106	RIKEN cDNA 1110035D15 gene(1110035D15Rik)	-1.69
BC005561	cDNA sequence BC005561(BC005561)	-1.69
NM_009056	regulatory factor X, 2 (influences HLA class II expression)(Rfx2)	-1.69
NM_020046	dihydroorotate dehydrogenase(Dhodh)	-1.69
L06451	nonagouti(a)	-1.69
BC005577	GTPase, IMAP family member 4(Gimap4)	-1.69
AF078869	solute carrier family 22 (organic anion transporter), member 8(Slc22a8)	-1.69
AF287893	golgi associated PDZ and coiled-coil motif containing(Gopc)	-1.69
NM_013646	RAR-related orphan receptor alpha(Rora)	-1.68
NM_013655	chemokine (C-X-C motif) ligand 12(Cxcl12)	-1.68
NM_015763	lipin 1(Lpin1)	-1.68
AK016528	RIKEN cDNA 4932416J16 gene(4932416J16Rik)	-1.68
AF232828	neuro-oncological ventral antigen 1(Nova1)	-1.68
AF072794		-1.68
NM_007615	catenin (cadherin associated protein), delta 1(Ctnnd1)	-1.68
NM_007961	ets variant 6(Etv6)	-1.68
NM_009461	ubiquitin protein ligase E3 component n-recognin 1(Ubr1)	-1.68
NM_008428	potassium inwardly-rectifying channel, subfamily J, member 8(Kcnj8)	-1.68
U49723		-1.68
NM_019797	thyroid hormone receptor interactor 4(Trip4)	-1.68
BC025873	phosphatase and actin regulator 1(Phactr1)	-1.68
AF295105	ryanodine receptor 2, cardiac(Ryr2)	-1.68
NM_010757	v-maf musculoaponeurotic fibrosarcoma oncogene family, protein K (avian)(Mafk)	-1.68
NM_009200	solute carrier family 1 (high affinity aspartate/glutamate transporter), member 6(Slc1a6)	-1.68
AF194028	histidine-rich glycoprotein(Hrg)	-1.68
NM_008061	glucose-6-phosphatase, catalytic(G6pc)	-1.67
AJ250103		-1.67

NM_011070	prefoldin 2(Pfdn2)	-1.67
AK016935	RIKEN cDNA 4933426K07 gene(4933426K07Rik)	-1.67
AK009375	CD300 molecule like family member G(Cd300lg)	-1.67
M61046		-1.67
AK003561	FCF1 rRNA processing protein(Fcf1)	-1.67
NM_019561	endosulfine alpha(Ensa)	-1.67
NM_010455	homeobox A7(Hoxa7)	-1.67
NM_009536	tyrosine 3-monooxygenase/tryptophan 5-monooxygenase activation protein, epsilon polypeptide(Ywhae)	-1.67
NM_018877	SET domain, bifurcated 1(Setdb1)	-1.67
NM_029693	RIKEN cDNA 1700123K08 gene(1700123K08Rik)	-1.67
AJ132681		-1.67
AB048947	synaptotagmin XIII(Syt13)	-1.67
NM_025853	DSN1 homolog, MIS12 kinetochore complex component(Dsn1)	-1.67
NM_008379	karyopherin (importin) beta 1(Kpnb1)	-1.67
AK008234	glucosaminyl (N-acetyl) transferase 3, mucin type(Gcnt3)	-1.66
NM_010241	thymoma viral proto-oncogene 1 interacting protein(Aktip)	-1.66
U55594		-1.66
AK018615	intestine specific homeobox(Isx)	-1.66
NM_007796	cytotoxic T lymphocyte-associated protein 2 alpha(Ctla2a)	-1.66
NM_009217	somatostatin receptor 2(Sstr2)	-1.66
NM_007552	Bmi1 polycomb ring finger oncogene(Bmi1)	-1.66
NM_010088	prolactin family 8, subfamily a, member 2(Prl8a2)	-1.66
Z12527		-1.66
AJ278462	matrix metallopeptidase 1a (interstitial collagenase)(Mmp1a)	-1.66
AK011562	RIKEN cDNA C330021F23 gene(C330021F23Rik)	-1.66
AJ132673		-1.66
NM_019665	ADP-ribosylation factor-like 6(Arl6)	-1.66
NM_025968	prostaglandin reductase 1(Ptgr1)	-1.66

NM_007526	BarH-like homeobox 1(Barx1)	-1.66
AF297220	glia maturation factor, beta(Gmfb)	-1.66
AF051947	calcium channel, voltage-dependent, T type, alpha 1H subunit(Cacna1h)	-1.65
AK020281	membrane bound O-acyltransferase domain containing 1(Mboat1)	-1.65
NM_008302	heat shock protein 90 alpha (cytosolic), class B member 1(Hsp90ab1)	-1.65
NM_007440	arachidonate 12-lipoxygenase(Alox12)	-1.65
AF200357	pantothenate kinase 1(Pank1)	-1.65
L34052		-1.65
NM_008681	N-myc downstream regulated gene 1(Ndrg1)	-1.65
AF272844	secretoglobin, family 2B, member 20(Scgb2b20)	-1.65
Z12550		-1.65
AK021246	RIKEN cDNA C430039J01 gene(C430039J01Rik)	-1.65
AK006238	RIKEN cDNA 1700022F17 gene(1700022F17Rik)	-1.65
NM_008593	forkhead box D2(Foxd2)	-1.65
AK005141	phosphoinositide-3-kinase interacting protein 1(Pik3ip1)	-1.65
AF294617	6-phosphofructo-2-kinase/fructose-2,6-biphosphatase 3(Pfkfb3)	-1.65
NM_011668	ubiquitin protein ligase E3A(Ube3a)	-1.65
NM_019873	FK506 binding protein-like(Fkbpl)	-1.65
NM_009184	PTK6 protein tyrosine kinase 6(Ptk6)	-1.65
NM_019963	signal transducer and activator of transcription 2(Stat2)	-1.64
NM_010146	epilepsy, progressive myoclonic epilepsy, type 2 gene alpha(Epm2a)	-1.64
AF029746		-1.64
U37883	immunoglobulin kappa variable 4-53(Igkv4-53)	-1.64
NM_009844	CD19 antigen(Cd19)	-1.64
AF155354	ankyrin repeat and SOCS box-containing 3(Asb3)	-1.64
NM_019481	solute carrier family 13 (sodium/sulfate symporters), member 1(Slc13a1)	-1.64
NM_029460	vomeronasal 2, receptor, pseudogene 54(Vmn2r-ps54)	-1.64
AK019605		-1.64

AK004966	leucine rich repeat containing 3(Lrrc3)	-1.64
X15684	solute carrier family 2 (facilitated glucose transporter), member 2(Slc2a2)	-1.64
NM_019736	acyl-CoA thioesterase 9(Acot9)	-1.64
NM_009512	solute carrier family 27 (fatty acid transporter), member 5(Slc27a5)	-1.64
NM_015783	ISG15 ubiquitin-like modifier(Isg15)	-1.64
AK016329	RIKEN cDNA 4930579K19 gene(4930579K19Rik)	-1.64
AK016541	RIKEN cDNA 4932432N04 gene(4932432N04Rik)	-1.64
AF073882	myotubularin related protein 7(Mtmr7)	-1.64
AF283667	phosphoglycerate mutase 1(Pgam1)	-1.63
NM_011331	chemokine (C-C motif) ligand 12(Ccl12)	-1.63
NM_008665	myelin transcription factor 1(Myt1)	-1.63
NM_008042	formyl peptide receptor 3(Fpr3)	-1.63
NM_009139	chemokine (C-C motif) ligand 6(Ccl6)	-1.63
NM_019398	ribonuclease, RNase A family, 2B (liver, eosinophil-derived neurotoxin)(Rnase2b)	-1.63
AF159256	RAN, member RAS oncogene family(Ran)	-1.63
NM_026949	CCR4-NOT transcription complex, subunit 8(Cnot8)	-1.63
X02568		-1.63
NM_009077	ribosomal protein L18(Rpl18)	-1.63
D90225	pleiotrophin(Ptn)	-1.63
Z22043		-1.63
NM_019814	HIG1 domain family, member 1A(Higd1a)	-1.63
NM_008791	Purkinje cell protein 4(Pcp4)	-1.63
NM_009215	somatostatin(Sst)	-1.63
NM_011820	gamma-glutamyltransferase 5(Ggt5)	-1.63
X99994		-1.63
AJ278829	pyroglutamyl-peptidase I(Pgpep1)	-1.63
NM_011271	ribonuclease, RNase A family, 1 (pancreatic)(Rnase1)	-1.63
NM_020583	interferon-stimulated protein(Isg20)	-1.63

NM_007469	apolipoprotein C-I(Apoc1)	-1.62
NM_021273	creatine kinase, brain(Ckb)	-1.62
NM_013842	X-box binding protein 1(Xbp1)	-1.62
AF304306	hydroxysteroid (17-beta) dehydrogenase 11(Hsd17b11)	-1.62
NM_007515	solute carrier family 7 (cationic amino acid transporter, y+ system), member 3(Slc7a3)	-1.62
AF162224	angiopoietin-like 3(Angptl3)	-1.62
NM_008942	aminopeptidase puromycin sensitive(Npepps)	-1.62
AB024499	zinc finger homeodomain 4(Zfhx4)	-1.62
NM_009530	alpha thalassemia/mental retardation syndrome X-linked(Atrx)	-1.62
NM_009119	Sin3-associated polypeptide 18(Sap18)	-1.62
AK014147	RIKEN cDNA 3110039I08 gene(3110039I08Rik)	-1.62
NM_011676	unc-119 lipid binding chaperone(Unc119)	-1.62
NM_021554	methyltransferase like 9(Mettl9)	-1.62
AK013702		-1.62
NM_009211	SWI/SNF related, matrix associated, actin dependent regulator of chromatin, subfamily c, member 1(Smarcc1)	-1.62
NM_007427	agouti related neuropeptide(Agrp)	-1.62
L20509	chaperonin containing Tcp1, subunit 3 (gamma)(Cct3)	-1.62
AF367759	syntrophin, gamma 1(Sntg1)	-1.62
NM_011125	phospholipid transfer protein(Pltp)	-1.62
NM_025746	RIKEN cDNA 4933415F23 gene(4933415F23Rik)	-1.61
NM_013771	YME1-like 1 (S. cerevisiae)(Yme1l1)	-1.61
NM_009716	activating transcription factor 4(Atf4)	-1.61
AB064489		-1.61
BC015275	retinoic acid receptor responder (tazarotene induced) 2(Rarres2)	-1.61
NM_009973	casein alpha s2-like B(Csn1s2b)	-1.61
AK015561	neurolysin (metallopeptidase M3 family)(Nln)	-1.61
U26225		-1.61

NM_026011	ADP-ribosylation factor-like 8B(Arl8b)	-1.61
NM_011925	adhesion G protein-coupled receptor E5(Adgre5)	-1.61
NM_009509	villin 1(Vil1)	-1.61
AK020780	Yip1 domain family, member 6(Yipf6)	-1.60
NM_018784	ST3 beta-galactoside alpha-2,3-sialyltransferase 6(St3gal6)	-1.60
AK018453	RIKEN cDNA 8430432A02 gene(8430432A02Rik)	-1.60
U63146	retinol binding protein 4, plasma(Rbp4)	-1.60
NM_008764	tumor necrosis factor receptor superfamily, member 11b (osteoprotegerin)(Tnfrsf11b)	-1.60
NM_010722	lamin B2(Lmnb2)	-1.60
NM_010565	inhibin beta-C(Inhbc)	-1.60
NM_012007	cathepsin J(Ctsj)	-1.60
NM_008091	GATA binding protein 3(Gata3)	-1.60
AK008356	RIKEN cDNA 2010109A12 gene(2010109A12Rik)	-1.60
NM_023431	melanoma associated antigen (mutated) 1(Mum1)	-1.60
NM_016881	phosphomannomutase 2(Pmm2)	-1.60
NM_009145	neuroplastin(Nptn)	-1.60
NM_013905	hairy/enhancer-of-split related with YRPW motif-like(Heyl)	-1.60
NM_021542	potassium channel, subfamily K, member 5(Kcnk5)	-1.60
NM_013750	pleckstrin homology like domain, family A, member 3(Phlda3)	-1.60
AK013790	integrator complex subunit 6(Ints6)	-1.60
NM_007460	adaptor-related protein complex 3, delta 1 subunit(Ap3d1)	-1.60
NM_025467	gastrokine 2(Gkn2)	-1.60
NM_011135	CCR4-NOT transcription complex, subunit 7(Cnot7)	-1.60
AK011682	RIKEN cDNA 2610035D17 gene(2610035D17Rik)	-1.60
AF321300	fibroblast growth factor receptor-like 1(Fgfrl1)	-1.60
U49723	guanylate cyclase 2c(Gucy2c)	-1.60
NM_013814	UDP-N-acetyl-alpha-D-galactosamine:polypeptide N-acetylgalactosaminyltransferase 1(Galnt1)	-1.60
M92419	stefin A3(Stfa3)	-1.59

Z12195		-1.59
NM_015740	biogenesis of lysosomal organelles complex-1, subunit 1(Bloc1s1)	-1.59
M19904		-1.59
AB027128	polymerase (DNA directed), eta (RAD 30 related)(Polh)	-1.59
AF156890	growth differentiation factor 2(Gdf2)	-1.59
U48737	pre-mRNA processing factor 4B(Prpf4b)	-1.59
AK006705	Rpgrip1-like(Rpgrip1l)	-1.59
NM_011544	transcription factor 12(Tcf12)	-1.59
AK019706	listerin E3 ubiquitin protein ligase 1(Ltn1)	-1.59
NM_009841	CD14 antigen(Cd14)	-1.59
AY013774	protocadherin beta 13(Pcdhb13)	-1.59
NM_019715	potassium channel modulatory factor 1(Kcmf1)	-1.58
AI153368		-1.58
D10627	zinc finger protein 930(Zfp930)	-1.58
X83590	ribosomal protein L5(Rpl5)	-1.58
AF015213	Ig heavy chain Mem5-like(LOC641025)	-1.58
AK016446	RIKEN cDNA 4931409D07 gene(4931409D07Rik)	-1.58
NM_013601	msh homeobox 2(Msx2)	-1.58
M57975	calcium channel, voltage-dependent, L type, alpha 1D subunit(Cacna1d)	-1.58
AK017406	zinc finger and BTB domain containing 21(Zbtb21)	-1.58
NM_012018	centriolin(Cntrl)	-1.58
AF128260		-1.58
D12487	choline acetyltransferase(Chat)	-1.58
NM_010822	N-methylpurine-DNA glycosylase(Mpg)	-1.58
AF190449	AF4/FMR2 family, member 4(Aff4)	-1.58
AK005839	RIKEN cDNA 1700010K24 gene(1700010K24Rik)	-1.57
AK014458	zinc finger and BTB domain containing 9(Zbtb9)	-1.57
NM_008002	fibroblast growth factor 10(Fgf10)	-1.57

NM_008788	procollagen C-endopeptidase enhancer protein(Pcolce)	-1.57
NM_010581	CD47 antigen (Rh-related antigen, integrin-associated signal transducer)(Cd47)	-1.57
NM_020610	nuclear receptor interacting protein 3(Nrip3)	-1.57
AF291492		-1.57
AB059428	acyl-CoA synthetase medium-chain family member 1(Acsm1)	-1.57
NM_007966	even-skipped homeobox 1(Evx1)	-1.57
NM_008779	contactin 3(Cntn3)	-1.57
NM_008301	heat shock protein 2(Hspa2)	-1.57
NM_020025	UDP-Gal:betaGlcNAc beta 1,3-galactosyltransferase, polypeptide 2(B3galt2)	-1.57
NM_015779	elastase, neutrophil expressed(Elane)	-1.57
AF305211	solute carrier family 28 (sodium-coupled nucleoside transporter), member 3(Slc28a3)	-1.57
AB033759	solute carrier family 4, sodium bicarbonate cotransporter-like, member 10(Slc4a10)	-1.57
M11943	wingless-type MMTV integration site family, member 1(Wnt1)	-1.57
NM_008182	glutathione S-transferase, alpha 2 (Yc2)(Gsta2)	-1.57
NM_011551	upstream binding transcription factor, RNA polymerase I(Ubtf)	-1.57
AF204959	cytochrome P450, family 3, subfamily a, polypeptide 25(Cyp3a25)	-1.57
NM_013671	superoxide dismutase 2, mitochondrial(Sod2)	-1.57
NM_007753	carboxypeptidase A3, mast cell(Cpa3)	-1.57
NM_010191	farnesyl diphosphate farnesyl transferase 1(Fdft1)	-1.57
AB010353	zinc finger with KRAB and SCAN domains 3(Zkscan3)	-1.57
NM_009825	serine (or cysteine) peptidase inhibitor, clade H, member 1(Serpinh1)	-1.57
NM_009017	retinoic acid early transcript beta(Raet1b)	-1.57
AF145716	schwannomin interacting protein 1(Schip1)	-1.57
AK005920	synaptophysin-like 2(Sypl2)	-1.57
U49951	mitogen-activated protein kinase kinase kinase 19(Map3k19)	-1.57
NM_027205		-1.57
NM_013599	matrix metallopeptidase 9(Mmp9)	-1.56
NM_011877	protein tyrosine phosphatase, non-receptor type 21(Ptpn21)	-1.56

AK010935	cysteine and glycine-rich protein 2 binding protein(Csrp2bp)	-1.56
NM_010023	enoyl-Coenzyme A delta isomerase 1(Eci1)	-1.56
AK015582	RIKEN cDNA 4930477O15 gene(4930477O15Rik)	-1.56
NM_007602	calpain 5(Capn5)	-1.56
NM_019514	astrotactin 2(Astn2)	-1.56
U55504		-1.56
AJ235939		-1.56
NM_031192	renin 1 structural(Ren1)	-1.56
NM_009152	sema domain, immunoglobulin domain (Ig), short basic domain, secreted, (semaphorin) 3A(Sema3a)	-1.56
AF223576	nuclear transcription factor, X-box binding 1(Nfx1)	-1.56
U51459		-1.56
NM_026634	RIKEN cDNA A930018P22 gene(A930018P22Rik)	-1.56
NM_010281	gamma-glutamyl hydrolase(Ggh)	-1.56
NM_009231	son of sevenless homolog 1 (Drosophila)(Sos1)	-1.56
NM_010412	histone deacetylase 5(Hdac5)	-1.56
NM_010451	homeobox A2(Hoxa2)	-1.56
NM_009014	RAD51 paralog B(Rad51b)	-1.56
X65010		-1.56
NM_009802	carbonic anhydrase 6(Car6)	-1.56
NM_021340	retinal G protein coupled receptor(Rgr)	-1.55
NM_008674	N-acetyltransferase 3(Nat3)	-1.55
NM_013552	hyaluronan mediated motility receptor (RHAMM)(Hmmr)	-1.55
NM_021313	ring finger protein 25(Rnf25)	-1.55
NM_013591	mucosal vascular addressin cell adhesion molecule 1(Madcam1)	-1.55
NM_010170	coagulation factor II (thrombin) receptor-like 2(F2rl2)	-1.55
NM_010670	keratin associated protein 12-1(Krtap12-1)	-1.55
NM_008054	Fyn proto-oncogene(Fyn)	-1.55
NM_021499	WD repeat containing, antisense to Trp73(Wrap73)	-1.55

AB041656	SS nuclear autoantigen 1(Ssna1)	-1.55
AF123386	zinc finger, MYND domain containing 10(Zmynd10)	-1.55
AJ409505		-1.55
NM_009885	carboxyl ester lipase(Cel)	-1.55
AJ400622	transmembrane protein with EGF-like and two follistatin-like domains 1(Tmeff1)	-1.55
NM_016775	DnaJ heat shock protein family (Hsp40) member C5(Dnajc5)	-1.55
NM_011703	vasoactive intestinal peptide receptor 1(Vipr1)	-1.55
AB047557	DEAH (Asp-Glu-Ala-His) box polypeptide 30(Dhx30)	-1.55
D45850	aldo-keto reductase family 1, member C6(Akr1c6)	-1.55
NM_020260	Rho GTPase activating protein 31(Arhgap31)	-1.55
AF111166	ryanodine receptor 3(Ryr3)	-1.55
AF361939	chymase 2, mast cell(Cma2)	-1.55
U89741		-1.55
M28730	tubulin, beta 4A class IVA(Tubb4a)	-1.55
NM_008313	5 hydroxytryptamine (serotonin) receptor 4(Htr4)	-1.55
AK008000	alkaline phosphatase, intestinal(Alpi)	-1.55
NM_021607	nicastrin(Ncstn)	-1.55
AK020244	chromodomain helicase DNA binding protein 9(Chd9)	-1.55
NM_011294	SUB1 homolog (S. cerevisiae)(Sub1)	-1.54
NM_026108	haloacid dehalogenase-like hydrolase domain containing 1A(Hdhd1a)	-1.54
NM_009063	regulator of G-protein signaling 5(Rgs5)	-1.54
NM_020013	fibroblast growth factor 21(Fgf21)	-1.54
NM_009182	ST8 alpha-N-acetyl-neuraminide alpha-2,8-sialyltransferase 3(St8sia3)	-1.54
AK015104	RIKEN cDNA 4930406D14 gene(4930406D14Rik)	-1.54
D00622	low density lipoprotein receptor-related protein associated protein 1(Lrpap1)	-1.54
AF289487	aspartate-beta-hydroxylase(Asph)	-1.54
AF059259		-1.54
AK015905	RIKEN cDNA 4930526L06 gene(4930526L06Rik)	-1.54

NM_013912	apelin(Apln)	-1.54
NM_009295	syntaxin binding protein 1(Stxbp1)	-1.54
Z12414		-1.54
AK020681	phosphatase and actin regulator 1(Phactr1)	-1.54
AK002441	transmembrane protein 254c(Tmem254c, b, a)	-1.54
NM_008236	hairy and enhancer of split 2 (Drosophila)(Hes2)	-1.54
NM_019721	methyltransferase like 3(Mettl3)	-1.53
NM_009320	solute carrier family 6 (neurotransmitter transporter, taurine), member 6(Slc6a6)	-1.53
AK017759	neuron-derived neurotrophic factor(Ndnf)	-1.53
BC005638	family with sequence similarity 220, member A(Fam220a)	-1.53
AF133734		-1.53
AK005419	cytochrome b-561 domain containing 1(Cyb561d1)	-1.53
AB010363	regulator of sex-limitation candidate 18(Rslcan18)	-1.53
NM_008225	hematopoietic cell specific Lyn substrate 1(Hcls1)	-1.53
J04847	DNA segment, Chr 1, Pasteur Institute 1(D1Pas1)	-1.53
NM_008419		-1.53
AF144397	carnitine O-octanoyltransferase(Crot)	-1.53
AK005772	t-complex 11 family, X-linked 2(Tcp11x2)	-1.53
NM_016671	interleukin 27 receptor, alpha(Il27ra)	-1.53
NM_008244	HGF-regulated tyrosine kinase substrate(Hgs)	-1.53
AF220156		-1.52
NM_010761	cyclin D-type binding-protein 1(Ccndbp1)	-1.52
AK012657	ectonucleoside triphosphate diphosphohydrolase 7(Entpd7)	-1.52
BC006187	cartilage intermediate layer protein 2(Cilp2)	-1.52
X68884	orthodenticle homeobox 2(Otx2)	-1.52
AK011476	mitochondrial ribosomal protein S30(Mrps30)	-1.52
AF054819	killer cell lectin-like receptor subfamily K, member 1(Klrk1)	-1.52
NM_011731	solute carrier family 6 (neurotransmitter transporter), member 20B(Slc6a20b)	-1.52

AJ307017	ubiquitin specific peptidase 9, Y chromosome(Usp9y)	-1.52
AJ238004	protein kinase, X-linked(Prkx)	-1.52
L11333	carboxylesterase 3A(Ces3a)	-1.52
NM_007860	deiodinase, iodothyronine, type I(Dio1)	-1.52
NM_007781	colony stimulating factor 2 receptor, beta 2, low-affinity (granulocyte-macrophage)(Csf2rb2)	-1.52
NM_013705	zinc finger protein 30(Zfp30)	-1.52
AK018334		-1.52
AB010364	zinc finger protein 748(Zfp748)	-1.52
Z78147		-1.51
AF131197	Eph receptor A1(Epha1)	-1.51
NM_029583		-1.51
AK006998	RIKEN cDNA 1700084M22 gene(1700084M22Rik)	-1.51
NM_012061	Ca2+-dependent secretion activator(Cadps)	-1.51
NM_028186	naked cuticle 2 homolog (Drosophila)(Nkd2)	-1.51
D50060	proprotein convertase subtilisin/kexin type 6(Pcsk6)	-1.51
NM_020575	membrane-associated ring finger (C3HC4) 7(March7)	-1.51
D87521	protein kinase, DNA activated, catalytic polypeptide(Prkdc)	-1.51
X90884		-1.51
NM_019922	cartilage associated protein(Crtap)	-1.51
AK005003	PX domain containing 1(Pxdc1)	-1.51
NM_009298	surfeit gene 6(Surf6)	-1.51
NM_009900	chloride channel, voltage-sensitive 2(Clcn2)	-1.51
NM_011960	poly (ADP-ribose) glycohydrolase(Parg)	-1.51
NM_016889	insulinoma-associated 1(Insm1)	-1.51
BC002228		-1.51
NM_019547	RNA binding motif protein 38(Rbm38)	-1.51
NM_008998	RAB17, member RAS oncogene family(Rab17)	-1.50
M93980	ribosomal protein L10(Rpl10)	-1.50

NM_008804	phosphodiesterase 9A(Pde9a)	-1.50
X80464		-1.50
D50389		-1.50
X17165		-1.50
NM_031389	NLR family, pyrin domain containing 4C(Nlrp4c)	-1.50
NM_007662	cadherin 15(Cdh15)	-1.50
M92378	solute carrier family 6 (neurotransmitter transporter, GABA), member 1(Slc6a1)	-1.50
AK014834	thioesterase superfamily member 4(Them4)	1.5
NM_018873	SRC kinase signaling inhibitor 1(Srcin1)	1.5
NM_030713	zinc finger protein 202(Zfp202)	1.5
Z12520		1.5
AF309132		1.5
AK017147	RIKEN cDNA 5033403F01 gene(5033403F01Rik)	1.5
NM_031166	inhibitor of DNA binding 4(Id4)	1.5
AK019098	RIKEN cDNA 2310063J23 gene(2310063J23Rik)	1.5
AK016058		1.5
NM_013621	olfactory receptor 69(Olfr69)	1.5
BC005511	tubulin tyrosine ligase(Ttl)	1.5
Z22132		1.5
AK015753	WD repeat domain 64(Wdr64)	1.5
AK016943	predicted gene 9757(Gm9757)	1.5
NM_020609	cDNA sequence BC051019(BC051019)	1.5
NM_011189	proteasome (prosome, macropain) activator subunit 1 (PA28 alpha)(Psme1)	1.5
NM_012017	zinc finger protein 346(Zfp346)	1.5
BC006747	Rho guanine nucleotide exchange factor (GEF) 10-like(Arhgef10l)	1.5
AK020182	zinc finger and BTB domain containing 20(Zbtb20)	1.5
NM_008679	nuclear receptor coactivator 3(Ncoa3)	1.5
AF050423		1.5

AK018143	cingulin(Cgn)	1.5
BC004773	tetratricopeptide repeat domain 30A1(Ttc30a1)	1.5
AK015443	RIKEN cDNA 4930451G21 gene(4930451G21Rik)	1.5
X79788		1.5
NM_024171	Sec61 beta subunit(Sec61b)	1.5
AK019760	methylcrotonoyl-Coenzyme A carboxylase 2 (beta)(Mccc2)	1.5
AB036742	ectopic ossification 1(Etos1)	1.5
AJ279852		1.5
AK012199	zinc finger, HIT domain containing 1(Znhit1)	1.5
AJ005844	tenascin R(Tnr)	1.5
AK006257	single-pass membrane protein with coiled-coil domains 2(Smco2)	1.5
AK007181	transmembrane protein 165(Tmem165)	1.5
AK004394	LRRN4 C-terminal like(Lrrn4cl)	1.5
NM_022884	betaine-homocysteine methyltransferase 2(Bhmt2)	1.5
AK016863	RIKEN cDNA 4933421H12 gene(4933421H12Rik)	1.5
NM_015784	periostin, osteoblast specific factor(Postn)	1.5
AK017148	RIKEN cDNA 5033403H07 gene(5033403H07Rik)	1.5
U26230		1.5
AK020239	RIKEN cDNA 8530402H02 gene(8530402H02Rik)	1.5
AK020409	T-box 20(Tbx20)	1.5
AK003907	RIKEN cDNA 1110021P09 gene(1110021P09Rik)	1.5
AY030275	nucleolar protein interacting with the FHA domain of MKI67(Nifk)	1.5
BC008239	t-complex 11 (mouse) like 2(Tcp11l2)	1.5
AJ278735	coenzyme Q8A(Coq8a)	1.5
M34857	homeobox B9(Hoxb9)	1.5
Z12294		1.5
NM_021716	fidgetin(Fign)	1.5
NM_016718	ninjurin 2(Ninj2)	1.5

NM_021393	negative elongation factor complex member B(Nelfb)	1.5
AK020911	KN motif and ankyrin repeat domains 1(Kank1)	1.5
NM_026656	mucolipin 2(Mcoln2)	1.5
AF269248	hemogen(Hemgn)	1.5
AK006015	RIKEN cDNA 1700016C19 gene(1700016C19Rik)	1.5
AK015307	RIKEN cDNA 4930434B07 gene(4930434B07Rik)	1.5
BC006944	ectonucleotide pyrophosphatase/phosphodiesterase 3(Enpp3)	1.5
X66903	filamin binding LIM protein 1(Fblim1)	1.5
AF316989		1.5
AJ308965	PC4 and SFRS1 interacting protein 1(Psip1)	1.5
AK016646	AT hook containing transcription factor 1(Ahctf1)	1.5
AK003416	integrin alpha V(Itgav)	1.5
Z25469	protein S (alpha)(Pros1)	1.5
AY042192	MAS-related GPR, member A2B(Mrgpra2b)	1.5
AK016032	RIKEN cDNA 4930543N07 gene(4930543N07Rik)	1.5
BC008101	RAS-like, family 11, member B(Rasl11b)	1.5
AK013479	RIKEN cDNA 2900003A17 gene(2900003A17Rik)	1.5
AK019578	CUGBP, Elav-like family member 3(Celf3)	1.5
NM_025695	structural maintenance of chromosomes 6(Smc6)	1.5
AK020397	interleukin-1 receptor-associated kinase 4(Irak4)	1.5
AK014129	RIKEN cDNA 3110037B15 gene(3110037B15Rik)	1.5
AK018267	mediator complex subunit 13-like(Med13l)	1.5
AF001866		1.5
AK011345	family with sequence similarity 64, member A(Fam64a)	1.5
L23164		1.5
AK006623	RIKEN cDNA 1700037J18 gene(1700037J18Rik)	1.5
NM_024255	hydroxysteroid dehydrogenase like 2(Hsdl2)	1.5
AK012983	CDC42 small effector 2(Cdc42se2)	1.5

AK017720	RIKEN cDNA 5730492I20 gene(5730492I20Rik)	1.5
AK015197	protein tyrosine phosphatase, receptor type, N polypeptide 2(Ptprn2)	1.5
NM_024268		1.5
AK020359	fetal and adult testis expressed 1(Fate1)	1.5
AK017241	RIKEN cDNA 5330417C22 gene(5330417C22Rik)	1.5
NM_016673	ciliary neurotrophic factor receptor(Cntfr)	1.5
AK014856		1.5
Z12403		1.5
NM_018733	sodium channel, voltage-gated, type I, alpha(Scn1a)	1.5
AF286212	armadillo repeat gene deleted in velo-cardio-facial syndrome(Arvcf)	1.5
AK008081	solute carrier protein 52, member 2(Slc52a2)	1.5
BC006682	eukaryotic translation initiation factor 2, subunit 3, structural gene X-linked(Eif2s3x)	1.5
AK007576	RIKEN cDNA 1810021M19 gene(1810021M19Rik)	1.5
AK003278	solute carrier family 46, member 1(Slc46a1)	1.5
AK019635	RIKEN cDNA 4930459L07 gene(4930459L07Rik)	1.5
AF290967		1.5
NM_030687	solute carrier organic anion transporter family, member 1a4(Slco1a4)	1.5
BC003937	erythrocyte membrane protein band 4.1 like 5(Epb41l5)	1.5
AJ231227		1.5
U28769		1.5
AK005451	endogenous retroviral sequence 3(Erv3)	1.5
NM_030697	KN motif and ankyrin repeat domains 3(Kank3)	1.5
BC005528	TRAF-interacting protein with forkhead-associated domain, family member B(Tifab)	1.5
NM_018867	carboxypeptidase X 2 (M14 family)(Cpxm2)	1.5
AK019642	RIKEN cDNA 4930471C04 gene(4930471C04Rik)	1.5
AK012412	RIKEN cDNA 2700052C19 gene(2700052C19Rik)	1.5
AK013447	RIKEN cDNA 2810487C13 gene(2810487C13Rik)	1.5
AK006094	RIKEN cDNA 1700018M17 gene(1700018M17Rik)	1.5

AK005974	RIKEN cDNA 1700014D04 gene(1700014D04Rik)	1.5
Z12248		1.5
AK020556	RIKEN cDNA 9530014B07 gene(9530014B07Rik)	1.5
U55496		1.5
NM_008612	menage a trois 1(Mnat1)	1.5
AJ231226		1.5
NM_013661	sema domain, seven thrombospondin repeats (type 1 and type 1-like), transmembrane domain (TM) and short cytoplasmic domain, (semaphorin) 5B(Sema5b)	1.5
M94302		1.5
K02245	prolactin family 2, subfamily c, member 2(Prl2c2)	1.5
NM_025934	RIO kinase 2 (yeast)(Riok2)	1.5
AK020718	RIKEN cDNA A230106O10 gene(A230106O10Rik)	1.5
U55681		1.5
AK006689	RIKEN cDNA 1700042O05 gene(1700042O05Rik)	1.5
NM_011433		1.5
NM_019865	ribosomal protein L36A(Rpl36a)	1.5
AK018440	abl-interactor 2(Abi2)	1.5
BC004717	unc-45 myosin chaperone A(Unc45a)	1.5
AK009156	TEN1 telomerase capping complex subunit(Ten1)	1.5
AK014159	RIKEN cDNA 3110040K02 gene(3110040K02Rik)	1.6
AF052218		1.6
U26768		1.6
AF240178		1.6
NM_025578	mitochondrial ribosomal protein S25(Mrps25)	1.6
AK006362	RIKEN cDNA 1700025O08 gene(1700025O08Rik)	1.6
BC011428	UDP-N-acetyl-alpha-D-galactosamine:polypeptide N-acetylgalactosaminyltransferase 11(Galnt11)	1.6
AB059656	nephronectin(Npnt)	1.6
Y13554		1.6

AK006219	RIKEN cDNA 1700021L22 gene(1700021L22Rik)	1.6
M92328		1.6
AK010539	Luc7-like(Luc7l)	1.6
AK020646	lysine (K)-specific methyltransferase 2E(Kmt2e)	1.6
NM_011856	teneurin transmembrane protein 2(Tenm2)	1.6
NM_013627	paired box 6(Pax6)	1.6
BC002137	cytochrome C oxidase assembly factor 5(Coa5)	1.6
NM_023907	forkhead box I1(Foxi1)	1.6
AK008825	Fas associated factor family member 2(Faf2)	1.6
AK004130	neurexophilin 4(Nxph4)	1.6
NM_008490	lecithin cholesterol acyltransferase(Lcat)	1.6
AK015957	RIKEN cDNA 4930533L02 gene(4930533L02Rik)	1.6
AK019788	syntaxin binding protein 5 (tomosyn)(Stxbp5)	1.6
BC004051		1.6
AK018099	RIKEN cDNA 6230426I18 gene(6230426I18Rik)	1.6
AK016117	RIKEN cDNA 4930554G03 gene(4930554G03Rik)	1.6
AK014380	PHD finger protein 19(Phf19)	1.6
NM_008526		1.6
AK012244	RIKEN cDNA 3300002A11 gene(3300002A11Rik)	1.6
NM_025520	LSM5 homolog, U6 small nuclear RNA and mRNA degradation associated(Lsm5)	1.6
AK020589	CSA-conditional, T cell activation-dependent protein(Cstad)	1.6
NM_019474	olfactory receptor 156(Olfr156)	1.6
U06945		1.6
AA733351		1.6
AK007391	ubiquitin protein ligase E3 component n-recognin 4(Ubr4)	1.6
NM_023133	ribosomal protein S19(Rps19)	1.6
NM_013594	methyl-CpG binding domain protein 1(Mbd1)	1.6
AK012172	coiled-coil domain containing 25(Ccdc25)	1.6

AJ297131		1.6
AK014890	REST corepressor 3(Rcor3)	1.6
AK015180	RIKEN cDNA 4930422I22 gene(4930422I22Rik)	1.6
M76131	eukaryotic translation elongation factor 2(Eef2)	1.6
AK020125	RIKEN cDNA 6720454L07 gene(6720454L07Rik)	1.6
AK007237	RIKEN cDNA 1700122E12 gene(1700122E12Rik)	1.6
Z18961	SRY (sex determining region Y)-box 12(Sox12)	1.6
L11065	opioid receptor, kappa 1(Oprk1)	1.6
BC003265		1.6
AK013430	RIKEN cDNA 2810481J17 gene(2810481J17Rik)	1.6
AK015576	RIKEN cDNA 4930474M22 gene(4930474M22Rik)	1.6
AK006840	THO complex 5(Thoc5)	1.6
NM_013669	synaptosomal-associated protein 91(Snap91)	1.6
AK019602	RIKEN cDNA 4930439D14 gene(4930439D14Rik)	1.6
AK013716	pluripotency-associated noncoding transcript 1(Panct1)	1.6
AK019439	RIKEN cDNA 3110062M04 gene(3110062M04Rik)	1.6
AK007683	C1q and tumor necrosis factor related protein 2(C1qtnf2)	1.6
AK018998	thymosin beta 15a(Tmsb15a)	1.6
NM_026454	ubiquitin-conjugating enzyme E2F (putative)(Ube2f)	1.6
BC006859	scribbled planar cell polarity(Scrib)	1.6
AK020637	RIKEN cDNA 9530071P10 gene(9530071P10Rik)	1.6
AF143541	CD55 molecule, decay accelerating factor for complement(Cd55)	1.6
AK013809	RIKEN cDNA 2900082C11 gene(2900082C11Rik)	1.6
AK006699	RIKEN cDNA 1700045I11 gene(1700045I11Rik)	1.6
L41881	immunoglobulin kappa variable 6-20(Igkv6-20)	1.6
AF018262	protein phosphatase 5, catalytic subunit(Ppp5c)	1.6
AK016369	testis expressed 26(Tex26)	1.6
AK019103	RIKEN cDNA 2400006E01 gene(2400006E01Rik)	1.6

AK016849	transformed mouse 3T3 cell double minute 4(Mdm4)	1.6
AK003661	zinc finger, FYVE domain containing 21(Zfyve21)	1.6
AK003555	shugoshin-like 2a (S. pombe)(Sgol2a)	1.6
NM_028311		1.6
AK015134	leucine rich repeat containing 52(Lrrc52)	1.6
NM_009016	retinoic acid early transcript 1, alpha(Raet1a)	1.6
AK011986	zinc finger protein 979(Zfp979)	1.6
AK007964	choline phosphotransferase 1(Chpt1)	1.6
AK021124	dopamine receptor D1(Drd1)	1.6
NM_028218		1.6
BC007151	NOP2 nucleolar protein(Nop2)	1.6
X80424	FAD-dependent oxidoreductase domain containing 1(Foxred1)	1.6
AK011997	RIKEN cDNA 2610306M01 gene(2610306M01Rik)	1.6
AK019750	RIKEN cDNA 4930548G05 gene(4930548G05Rik)	1.6
AK007988	cadherin-related family member 5(Cdhr5)	1.6
NM_030724	uridine-cytidine kinase 2(Uck2)	1.6
AY030406	PAK1 interacting protein 1(Pak1ip1)	1.6
AK020727	prune homolog 2(Prune2)	1.6
BC015299	adenylate cyclase 4(Adcy4)	1.6
AK019688	RIKEN cDNA 4930520K02 gene(4930520K02Rik)	1.6
AK003613	transmembrane p24 trafficking protein 4(Tmed4)	1.6
NM_016766	microspherule protein 1(Mcrs1)	1.6
NM_010922	mitochondrial ribosomal protein L40(Mrpl40)	1.6
Z68183	thymoma viral proto-oncogene 1 interacting protein(Aktip)	1.6
AK018729	RIKEN cDNA 0610008J02 gene(0610008J02Rik)	1.6
BB258473		1.6
NM_021428	dexamethasone-induced transcript(Dexi)	1.6
NM_013643	protein tyrosine phosphatase, non-receptor type 5(Ptpn5)	1.6

AK007096	RIKEN cDNA 1700069L16 gene(1700069L16Rik)	1.6
Y11811		1.6
AK014466	kelch-like 28(Klhl28)	1.6
AK018741	AAR2 splicing factor homolog(Aar2)	1.6
NM_025605	late endosomal/lysosomal adaptor, MAPK and MTOR activator 1(Lamtor1)	1.6
AK020060	pellino 3(Peli3)	1.6
X83886	vesicle-associated membrane protein 1(Vamp1)	1.6
AK015734	RIKEN cDNA 4930509E22 gene(4930509E22Rik)	1.6
AF317900	lactamase, beta(Lactb)	1.6
AK016270	RIKEN cDNA 4930430F08 gene(4930430F08Rik)	1.6
AK012914	leucine rich repeat protein 1, neuronal(Lrrn1)	1.6
AK015974	RIKEN cDNA 4930535B17 gene(4930535B17Rik)	1.6
AK007305	RIKEN cDNA 1700128A07 gene(1700128A07Rik)	1.6
AK020860	RIKEN cDNA A930015D03 gene(A930015D03Rik)	1.6
BC002251	phosphoserine phosphatase(Psph)	1.6
NM_028513	actin-related protein T2(Actrt2)	1.6
U21448		1.6
AK016442	RIKEN cDNA 4931407J08 gene(4931407J08Rik)	1.6
AK005229	mitochondrial ribosomal protein S28(Mrps28)	1.6
AK013951	protein phosphatase 4, regulatory subunit 1(Ppp4r1)	1.6
AK004153	guanylyl cyclase domain containing 1(Gucd1)	1.6
BC004768	Holliday junction recognition protein(Hjurp)	1.6
BC006733	WW, C2 and coiled-coil domain containing 1(Wwc1)	1.6
AF012171		1.6
AK019053	predicted gene 15247(Gm15247)	1.6
X51847		1.6
AK014964	SLAIN motif family, member 1, opposite strand(Slain1os)	1.6
AK010113	RIKEN cDNA 2310068J16 gene(2310068J16Rik)	1.6

AK006096	major facilitator superfamily domain containing 2A(Mfsd2a)	1.6
AK013843	RIKEN cDNA 2900092N22 gene(2900092N22Rik)	1.6
NM_019464	SH3-domain GRB2-like B1 (endophilin)(Sh3glb1)	1.6
NM_010672	keratin associated protein 6-1(Krtap6-1)	1.6
AK018053	RIKEN cDNA 5830474E16 gene(5830474E16Rik)	1.6
M27754		1.6
NM_021505	anaphase-promoting complex subunit 5(Anapc5)	1.6
NM_025315	mediator complex subunit 21(Med21)	1.6
U07950	guanosine diphosphate (GDP) dissociation inhibitor 1(Gdi1)	1.6
NM_026321	family with sequence similarity 174, member A(Fam174a)	1.6
AK020865	RIKEN cDNA A930015P04 gene(A930015P04Rik)	1.6
AY042193	MAS-related GPR, member A3(Mrgpra3)	1.6
AK021005	AT rich interactive domain 1B (SWI-like)(Arid1b)	1.6
U88067		1.6
AK004346	amylo-1,6-glucosidase, 4-alpha-glucanotransferase(Agl)	1.6
NM_007432	alkaline phosphatase 3, intestine, not Mn requiring(Akp3)	1.6
AK012323		1.6
AK006767	amyotrophic lateral sclerosis 2 (juvenile) chromosome region, candidate 11 (human)(Als2cr11)	1.6
AK006822	RIKEN cDNA 1700057D03 gene(1700057D03Rik)	1.6
NM_009421	TNF receptor-associated factor 1(Traf1)	1.6
AK004319	ELOVL family member 5, elongation of long chain fatty acids (yeast)(Elovl5)	1.6
AK010031	suppressor of cytokine signaling 7(Socs7)	1.6
AK004111	RIKEN cDNA 1110035H17 gene(1110035H17Rik)	1.6
AK015932	RIKEN cDNA 4930529F21 gene(4930529F21Rik)	1.6
AK015998	RIKEN cDNA 4930563I02 gene(4930563I02Rik)	1.6
AK007284	RIKEN cDNA 1700125M20 gene(1700125M20Rik)	1.6
AK015066	RIKEN cDNA 4930403L11 gene(4930403L11Rik)	1.6
AK005628	RIKEN cDNA 1700003E16 gene(1700003E16Rik)	1.6

AK018572	RIKEN cDNA 9030624G23 gene(9030624G23Rik)	1.6
NM_007787		1.6
AF294825		1.6
NM_026531	apoptosis enhancing nuclease(Aen)	1.6
AK020519	RIKEN cDNA 9430092D12 gene(9430092D12Rik)	1.6
Z12477		1.6
AK006900	RIKEN cDNA 1700066B19 gene(1700066B19Rik)	1.6
AK021240	RIKEN cDNA C430014O12 gene(C430014O12Rik)	1.6
NM_010232	flavin containing monooxygenase 5(Fmo5)	1.6
NM_009238	SRY (sex determining region Y)-box 4(Sox4)	1.6
NM_026096	huntingtin interacting protein M(Hypm)	1.6
AF041891		1.6
AK018176	serine/arginine-rich splicing factor 1(Srsf1)	1.6
NM_010150	nuclear receptor subfamily 2, group F, member 6(Nr2f6)	1.6
AK020378	RIKEN cDNA 9330161L09 gene(9330161L09Rik)	1.6
AK003991	nudix (nucleoside diphosphate linked moiety X)-type motif 14(Nudt14)	1.6
AK021338	RIKEN cDNA D730044K07 gene(D730044K07Rik)	1.6
AK011469	FERM domain containing 6(Frmd6)	1.6
AK016932	RIKEN cDNA 4933426I03 gene(4933426I03Rik)	1.6
BC004079	mercaptopyruvate sulfurtransferase(Mpst)	1.6
AK005212	nuclear export mediator factor(Nemf)	1.6
AK016814	RIKEN cDNA 4933415A04 gene(4933415A04Rik)	1.6
Z12396		1.6
NM_025687	testis expressed gene 12(Tex12)	1.6
AK013190	SWI/SNF related, matrix associated, actin dependent regulator of chromatin, subfamily c, member 2(Smarcc2)	1.6
AB030199	integral membrane protein 2C(Itm2c)	1.6
NM_026119	mediator complex subunit 4(Med4)	1.6

NM_008486	alanyl (membrane) aminopeptidase(Anpep)	1.6
U48240	D4, zinc and double PHD fingers family 1(Dpf1)	1.6
BC011059	vacuolar protein sorting 33B(Vps33b)	1.6
AK007402	cell growth regulator with ring finger domain 1(Cgrrf1)	1.6
NM_013517	Fc receptor, IgE, low affinity II, alpha polypeptide(Fcer2a)	1.6
NM_023173	dual specificity phosphatase 12(Dusp12)	1.6
X53630		1.6
Z11664	son of sevenless homolog 2 (Drosophila)(Sos2)	1.6
AK003642	RIKEN cDNA 1110012L19 gene(1110012L19Rik)	1.6
AF038572	jagged 2(Jag2)	1.6
Z86006	T cell receptor beta, joining region(Tcrb-J)	1.6
AK011940	RIKEN cDNA 2610300A13 gene(2610300A13Rik)	1.6
NM_025404	ADP-ribosylation factor-like 4D(Arl4d)	1.6
BC002112		1.6
AK009217	kallikrein related-peptidase 12(Klk12)	1.6
NM_026693	gamma-aminobutyric acid (GABA) A receptor-associated protein-like 2(Gabarapl2)	1.6
NM_008934	protein C(Proc)	1.6
AK013682	RIKEN cDNA 2900054C01 gene(2900054C01Rik)	1.6
AK021308	RIKEN cDNA D530034E23 gene(D530034E23Rik)	1.6
NM_009608	actin, alpha, cardiac muscle 1(Actc1)	1.6
AK006658	protease-associated domain containing 1(Pradc1)	1.6
AK010427	RIKEN cDNA 2410006H16 gene(2410006H16Rik)	1.6
AK004108	RIKEN cDNA 1110035E04 gene(1110035E04Rik)	1.6
AK014684	RIKEN cDNA 4833412C15 gene(4833412C15Rik)	1.6
AK012120	solute carrier family 39 (zinc transporter), member 9(Slc39a9)	1.6
NM_019460	Scm-like with four mbt domains 1(Sfmbt1)	1.7
AK019832	RIKEN cDNA 4930588G05 gene(4930588G05Rik)	1.7
NM_018769	deafness, autosomal dominant 5 (human)(Dfna5)	1.7

AK019758	RIKEN cDNA 4930551E15 gene(4930551E15Rik)	1.7
AK014627	RIKEN cDNA 4731417B20 gene(4731417B20Rik)	1.7
AK021254	whirlin(Whrn)	1.7
BC002226	hook microtubule tethering protein 2(Hook2)	1.7
AK014832	Down syndrome cell adhesion molecule like 1(Dscaml1)	1.7
AK020326	RIKEN cDNA 9230108I15 gene(9230108I15Rik)	1.7
AK011166	Scarletltr, erythroid developmental long intergenic non-protein coding transcript(Scarletltr)	1.7
AK004405	XRCC6 binding protein 1(Xrcc6bp1)	1.7
BC014728	transmembrane protein 38A(Tmem38a)	1.7
AK013967	glutathione S-transferase, C-terminal domain containing(Gstcd)	1.7
NM_011631	heat shock protein 90, beta (Grp94), member 1(Hsp90b1)	1.7
NM_008856	protein kinase C, eta(Prkch)	1.7
NM_007714	CDC like kinase 4(Clk4)	1.7
AK014048	RIKEN cDNA 3110013M02 gene(3110013M02Rik)	1.7
AK004569	cell division cycle 40(Cdc40)	1.7
AK020390	Scm-like with four mbt domains 1(Sfmbt1)	1.7
NM_020272	phosphoinositide-3-kinase, catalytic, gamma polypeptide(Pik3cg)	1.7
NM_026208	RIKEN cDNA 1700019N19 gene(1700019N19Rik)	1.7
AK005591	RIKEN cDNA 1700001G11 gene(1700001G11Rik)	1.7
NM_018775	TBC1 domain family, member 8(Tbc1d8)	1.7
AK008233	motile sperm domain containing 4(Mospd4)	1.7
NM_025639	centromere protein M(Cenpm)	1.7
AK013752	zinc finger and BTB domain containing 25(Zbtb25)	1.7
AK004989	mitochondrial amidoxime reducing component 1(Marc1)	1.7
AK020356	RIKEN cDNA 9230118H08 gene(9230118H08Rik)	1.7
AK007109	RIKEN cDNA 1700101O05 gene(1700101O05Rik)	1.7
AK021018	centlein, centrosomal protein(Cntln)	1.7
AK004067	inositol 1,4,5-trisphosphate 3-kinase B(Itpkb)	1.7

X94291	small nucleolar RNA, H/ACA box 74A(Snora74a)	1.7
AK007309	RIKEN cDNA 1700128E19 gene(1700128E19Rik)	1.7
AK019909	rabenosyn, RAB effector(Rbsn)	1.7
X16214		1.7
AK007076		1.7
NM_021474	epidermal growth factor-containing fibulin-like extracellular matrix protein 2(Efemp2)	1.7
AK018655	RIKEN cDNA 9130401M01 gene(9130401M01Rik)	1.7
AK013926	RIKEN cDNA 3100003L05 gene(3100003L05Rik)	1.7
NM_008865	prolactin family 3, subfamily b, member 1(Prl3b1)	1.7
AF187073		1.7
AK003700	WAS/WASL interacting protein family, member 2(Wipf2)	1.7
AJ132672		1.7
AK017006	RIKEN cDNA 4933431J24 gene(4933431J24Rik)	1.7
AB055854	predicted gene, 21951(Gm21951)	1.7
NM_009639	cysteine-rich secretory protein 3(Crisp3)	1.7
NM_021414	S-adenosylhomocysteine hydrolase-like 2(Ahcyl2)	1.7
AK019908	RIKEN cDNA 5330425B07 gene(5330425B07Rik)	1.7
BC004674	RNA binding motif protein 10(Rbm10)	1.7
AK013535	RIKEN cDNA 2900016D18 gene(2900016D18Rik)	1.7
AK008369	small nucleolar RNA host gene 8(Snhg8)	1.7
NM_016894	receptor (calcitonin) activity modifying protein 1(Ramp1)	1.7
NM_033072	methyl-CpG binding domain protein 6(Mbd6)	1.7
AK014067	scaffold attachment factor B(Safb)	1.7
AK017038	RIKEN cDNA 4933433F19 gene(4933433F19Rik)	1.7
NM_011530	transporter 2, ATP-binding cassette, sub-family B (MDR/TAP)(Tap2)	1.7
BC008534	TRAF3 interacting protein 2(Traf3ip2)	1.7
AK011926	RIKEN cDNA 2610209C05 gene(2610209C05Rik)	1.7
AK014557	tubulin tyrosine ligase-like family, member 4(Ttll4)	1.7

NM_026584	general transcription factor II E, polypeptide 2 (beta subunit)(Gtf2e2)	1.7
AF031635		1.7
AK016332	RIKEN cDNA 4930567H12 gene(4930567H12Rik)	1.7
NM_023231	stomatin (Epb7.2)-like 2(Stoml2)	1.7
L16799		1.7
AK010754	protein phosphatase 2, regulatory subunit A, beta(Ppp2r1b)	1.7
AK017905	RIKEN cDNA 5830407E08 gene(5830407E08Rik)	1.7
AK007899	RAP1 GTPase activating protein, opposite strand(Rap1gapos)	1.7
AK005986	RIKEN cDNA 1700015C15 gene(1700015C15Rik)	1.7
AK015166	coiled-coil domain containing 171(Ccdc171)	1.7
NM_016983	pre-B lymphocyte gene 2(Vpreb2)	1.7
AJ243933	cyclic nucleotide gated channel alpha 3(Cnga3)	1.7
NM_008123	gap junction protein, alpha 8(Gja8)	1.7
AK008890	zinc finger protein 84(Zfp84)	1.7
AK010332	Nanog homeobox(Nanog)	1.7
AK006976	RIKEN cDNA 1700082C01 gene(1700082C01Rik)	1.7
NM_026632	replication protein A3(Rpa3)	1.7
AK020885	spermatogenesis associated 9(Spata9)	1.7
NM_019428	ribonuclease P/MRP 30 subunit(Rpp30)	1.7
AK006973	RIKEN cDNA 1700081N11 gene(1700081N11Rik)	1.7
NM_009344	pleckstrin homology like domain, family A, member 1(Phlda1)	1.7
U43721	cystathionine beta-synthase(Cbs)	1.7
AK004439	adenylate kinase 8(Ak8)	1.7
AF000908		1.7
NM_026509	muscle-related coiled-coil protein(Murc)	1.7
NM_026267	NECAP endocytosis associated 1(Necap1)	1.7
AJ277219		1.7
NM_008624	muscle and microspikes RAS(Mras)	1.7

AK008069	polymerase (RNA) III (DNA directed) polypeptide G like(Polr3gl)	1.7
AF365932	zinc finger, imprinted 3(Zim3)	1.7
Z12491		1.7
BC002153	heat shock factor binding protein 1(Hsbp1)	1.7
AK021073	RIKEN cDNA C030013D06 gene(C030013D06Rik)	1.7
NM_026519	ER membrane protein complex subunit 4(Emc4)	1.7
BC006074	apoptosis resistant E3 ubiquitin protein ligase 1(Arel1)	1.7
AF042360	olfactory receptor 1355(Olfr1355)	1.7
NM_025469	colipase, pancreatic(Clps)	1.7
AK004331	peptidylprolyl isomerase (cyclophilin)-like 1(Ppil1)	1.7
U72677	oncoprotein induced transcript 1(Oit1)	1.7
NM_011963	pregnancy specific glycoprotein 18(Psg18)	1.7
AK021065	RIKEN cDNA C030011I16 gene(C030011I16Rik)	1.7
AK011386	myeloid/lymphoid or mixed-lineage leukemia; translocated to, 3(Mllt3)	1.7
AK017368	RIKEN cDNA 5430431A17 gene(5430431A17Rik)	1.7
AK019768	RIKEN cDNA 4930556A17 gene(4930556A17Rik)	1.7
NM_033042	tumor necrosis factor receptor superfamily, member 25(Tnfrsf25)	1.7
AF408433	transmembrane protein 209(Tmem209)	1.7
NM_007535	B cell leukemia/lymphoma 2 related protein A1c(Bcl2a1c)	1.7
AK013640		1.7
AK004409	membrane protein, palmitoylated 7 (MAGUK p55 subfamily member 7)(Mpp7)	1.7
AK021287	RIKEN cDNA D530014G21 gene(D530014G21Rik)	1.7
L16846	B cell translocation gene 1, anti-proliferative(Btg1)	1.7
NM_023311	Yip1 domain family, member 5(Yipf5)	1.7
AK002741	citrate lyase beta like(Clybl)	1.7
AF282286		1.7
AF093875		1.7
AK012608	cutA divalent cation tolerance homolog(Cuta)	1.7

NM_016985	myotubularin related protein 1(Mtmr1)	1.7
AK009861	zinc finger, CCHC domain containing 24(Zcchc24)	1.7
AK020834	RIKEN cDNA A930009A15 gene(A930009A15Rik)	1.7
NM_019933	protein tyrosine phosphatase, non-receptor type 4(Ptpn4)	1.7
S41227		1.7
AK013153	death associated protein kinase 1(Dapk1)	1.7
AJ250106	bicaudal D homolog 2 (Drosophila)(Bicd2)	1.7
AK014520	ATPase type 13A4(Atp13a4)	1.7
AK020300	cystatin 11(Cst11)	1.7
AK014419	nucleoporin 205(Nup205)	1.7
AK008165	kynurenine aminotransferase 1(Kyat1)	1.7
AK015930	zinc finger, BED domain containing 4 pseudogene(4930529C04Rik)	1.7
AK014831	RIKEN cDNA 4921507G05 gene(4921507G05Rik)	1.7
AF014451		1.7
AK014880	armadillo repeat containing 3(Armc3)	1.7
AK007279	RIKEN cDNA 1700125G22 gene(1700125G22Rik)	1.7
AK016050	RIKEN cDNA 4930546E12 gene(4930546E12Rik)	1.7
NM_025465	translation machinery associated 16(Tma16)	1.7
NM_010496	inhibitor of DNA binding 2(Id2)	1.7
NM_026124	RIKEN cDNA 1110008F13 gene(1110008F13Rik)	1.7
AK015158	family with sequence similarity 228, member A(Fam228a)	1.7
U57328	T-box 3(Tbx3)	1.7
AK019250	RIKEN cDNA 2810030D12 gene(2810030D12Rik)	1.7
AK012763	RIKEN cDNA 2810019C22 gene(2810019C22Rik)	1.7
AB031386	transmembrane protein 176B(Tmem176b)	1.7
AK021367	RIKEN cDNA E130102C15 gene(E130102C15Rik)	1.7
BC006931	expressed sequence AI597479(AI597479)	1.7
BF531396		1.7

AK013267	tetratricopeptide repeat domain 39C(Ttc39c)	1.7
AK020548	RIKEN cDNA 9530006O14 gene(9530006O14Rik)	1.7
AK014837	alkB homolog 3, opposite strand 1(Alkbh3os1)	1.7
Z12220		1.7
AK008216	mitochondrial calcium uniporter(Mcu)	1.7
AK016620	RIKEN cDNA 4933402J10 gene(4933402J10Rik)	1.7
AF394596	hypocretin (orexin) receptor 1(Hcrtr1)	1.7
AK008900	transmembrane protein 238(Tmem238)	1.7
Z78145	microtubule associated serine/threonine kinase family member 4(Mast4)	1.7
AK014697	dendrocyte expressed seven transmembrane protein(Dcstamp)	1.7
AK015917	dual specificity phosphatase 18(Dusp18)	1.7
AK008856	Ral GEF with PH domain and SH3 binding motif 2(Ralgps2)	1.8
BC002310	zinc finger protein 652(Zfp652)	1.8
D14175		1.8
BC011335	golgi-specific brefeldin A-resistance factor 1(Gbf1)	1.8
AK005716	EF-hand calcium binding domain 9(Efcab9)	1.8
AK021023	nucleolar protein 3 (apoptosis repressor with CARD domain)(Nol3)	1.8
NM_025290	radial spoke head 1 homolog (Chlamydomonas)(Rsph1)	1.8
M92329		1.8
BC010713	ADP-ribosylation factor related protein 1(Arfrp1)	1.8
AK020455	NF-kappaB repressing factor(Nkrf)	1.8
AK019718	RIKEN cDNA 4930534H18 gene(4930534H18Rik)	1.8
AK015682	RIKEN cDNA 4930502M04 gene(4930502M04Rik)	1.8
BC013625	NOL1/NOP2/Sun domain family member 2(Nsun2)	1.8
Z12211		1.8
AK013061	cat eye syndrome chromosome region, candidate 2(Cecr2)	1.8
NM_007674	caudal type homeobox 4(Cdx4)	1.8
AK008832	small integral membrane protein 24(Smim24)	1.8

AK017067	maestro(Mro)	1.8
NM_011770	IKAROS family zinc finger 2(Ikzf2)	1.8
X02569		1.8
U34960	guanine nucleotide binding protein (G protein), beta 2(Gnb2)	1.8
AF357488		1.8
BC004010	RIKEN cDNA 9430015G10 gene(9430015G10Rik)	1.8
AK008070	eukaryotic translation initiation factor 1A domain containing(Eif1ad)	1.8
AK013908	RIKEN cDNA 3021401N23 gene(3021401N23Rik)	1.8
AK006782	nipsnap homolog 3A (C. elegans)(Nipsnap3a)	1.8
U55527		1.8
AK010701	death effector domain-containing DNA binding protein 2(Dedd2)	1.8
AJ251685	glycoprotein (transmembrane) nmb(Gpnmb)	1.8
AK004546	osteopetrosis associated transmembrane protein 1(Ostm1)	1.8
AK020941	RIKEN cDNA A930037O16 gene(A930037O16Rik)	1.8
BC014755	eukaryotic translation initiation factor 3, subunit H(Eif3h)	1.8
BC013487	DnaJ heat shock protein family (Hsp40) member C17(Dnajc17)	1.8
AF338224		1.8
AK020437	Ts translation elongation factor, mitochondrial(Tsfm)	1.8
BC004580	RAB28, member RAS oncogene family(Rab28)	1.8
NM_030239	ATP-binding cassette, sub-family G (WHITE), member 3(Abcg3)	1.8
NM_025439	transmembrane protein 9(Tmem9)	1.8
NM_025403	NOP10 ribonucleoprotein(Nop10)	1.8
NM_016714	nucleoporin 50(Nup50)	1.8
M21383		1.8
NM_023557	solute carrier family 44, member 4(Slc44a4)	1.8
AF287293	LETM1 domain containing 1(Letmd1)	1.8
AK020345	RIKEN cDNA 9230113P08 gene(9230113P08Rik)	1.8
M33395		1.8

NM_019747	zinc finger protein 113(Zfp113)	1.8
AJ251363	torsin A interacting protein 2(Tor1aip2)	1.8
AK016348	RIKEN cDNA 4930584E12 gene(4930584E12Rik)	1.8
AK004374	RIKEN cDNA 1110065H08 gene(1110065H08Rik)	1.8
BC008274	paroxysmal nonkinesiogenic dyskinesia(Pnkd)	1.8
NM_011755	zinc finger protein 35(Zfp35)	1.8
AK016565	RIKEN cDNA 4932442E05 gene(4932442E05Rik)	1.8
AK013346	Kdm5c adjacent non-coding transcript(Kantr)	1.8
AK016142	RIKEN cDNA 4930556C24 gene(4930556C24Rik)	1.8
AK014261	RIKEN cDNA 3110079O15 gene(3110079O15Rik)	1.8
AK006797	IQ motif containing F1(Iqcf1)	1.8
Z12451		1.8
U29238		1.8
AK016483	RIKEN cDNA 4931428L18 gene(4931428L18Rik)	1.8
U15218	dystrophin, muscular dystrophy(Dmd)	1.8
AF106853	crystallin, beta B1(Crybb1)	1.8
AK017325	sarcoglycan, gamma (dystrophin-associated glycoprotein)(Sgcg)	1.8
NM_023182	chymotrypsin-like(Ctrl)	1.8
NM_025759	spermatogenesis associated glutamate (E)-rich protein 4D(Speer4d)	1.8
AF041882		1.8
NM_025523	NADH dehydrogenase (ubiquinone) 1, subcomplex unknown, 1(Ndufc1)	1.8
U76754	ring finger protein 213(Rnf213)	1.8
AK004796	RIKEN cDNA 1200015M12 gene(1200015M12Rik)	1.8
AK021118	regulating synaptic membrane exocytosis 1(Rims1)	1.8
NM_011172	proline dehydrogenase(Prodh)	1.8
AK004564	NHL repeat containing 2(Nhlrc2)	1.8
AK017086	RIKEN cDNA 4933436I20 gene(4933436I20Rik)	1.8
AK015013	rotatin(Rttn)	1.8

AK012049	family with sequence similarity 172, member A(Fam172a)	1.8
AF041960		1.8
U16180		1.8
NM_026042	mediator complex subunit 29(Med29)	1.8
AK016766	RIKEN cDNA 4933411D12 gene(4933411D12Rik)	1.8
Z12246		1.8
AK002641	nudix (nucleoside diphosphate linked moiety X)-type motif 12(Nudt12)	1.8
U55488		1.8
NM_013612	solute carrier family 11 (proton-coupled divalent metal ion transporters), member 1(Slc11a1)	1.8
AK014556	EPS8-like 1(Eps8l1)	1.8
NM_011824	gremlin 1, DAN family BMP antagonist(Grem1)	1.8
AF249870	PERP, TP53 apoptosis effector(Perp)	1.8
AK020225	RIKEN cDNA 8030497O21 gene(8030497O21Rik)	1.8
NM_026105	RIKEN cDNA 1700093K21 gene(1700093K21Rik)	1.8
BC007167	neurexophilin 3(Nxph3)	1.8
AK010163	transmembrane protein 123(Tmem123)	1.8
AK015160	RIKEN cDNA 4930417O22 gene(4930417O22Rik)	1.8
AK016682	RIKEN cDNA 4933406C10 gene(4933406C10Rik)	1.8
BC006063	nonhomologous end-joining factor 1(Nhej1)	1.8
NM_026522	chitinase domain containing 1(Chid1)	1.8
AK015313	potassium channel tetramerisation domain containing 16(Kctd16)	1.8
AK017571	cholinergic receptor, nicotinic, beta polypeptide 3(Chrnb3)	1.8
NM_008245	hematopoietically expressed homeobox(Hhex)	1.8
NM_016810	golgi SNAP receptor complex member 1(Gosr1)	1.8
AK013547	transmembrane protein 86B(Tmem86b)	1.8
NM_008135	solute carrier family 6 (neurotransmitter transporter, glycine), member 9(Slc6a9)	1.8
NM_008412	involucrin(Ivl)	1.8
AK020241	RIKEN cDNA 9030201C23 gene(9030201C23Rik)	1.8

NM_008524	lumican(Lum)	1.8
Z78156	DDHD domain containing 2(Ddhd2)	1.8
AK008228	farnesyltransferase, CAAX box, beta(Fntb)	1.8
AK006376	RIKEN cDNA 1700026D11 gene(1700026D11Rik)	1.8
L09105		1.8
AK006067	RIKEN cDNA 1700017I07 gene(1700017I07Rik)	1.8
NM_023120	guanine nucleotide binding protein (G protein), beta polypeptide 1-like(Gnb1l)	1.8
AK016661	RIKEN cDNA 4933405D12 gene(4933405D12Rik)	1.8
AF092039	steroid receptor RNA activator 1(Sra1)	1.8
AK017418	RIKEN cDNA 5430440P10 gene(5430440P10Rik)	1.8
AK015245	transmembrane protein 81(Tmem81)	1.8
AK012601	matrix-remodelling associated 7(Mxra7)	1.8
BC005546	ERBB receptor feedback inhibitor 1(Errfi1)	1.8
AB043357		1.8
NM_019979	selenoprotein K(Selk)	1.8
AK006872	RIKEN cDNA 1700063J08 gene(1700063J08Rik)	1.8
AK019166	spermatogenesis associated 2-like(Spata2l)	1.8
AK014727	RIKEN cDNA 4833419G08 gene(4833419G08Rik)	1.8
AK015658	RIKEN cDNA 4930500F04 gene(4930500F04Rik)	1.8
AK007940	prefoldin 4(Pfdn4)	1.8
NM_019917	vomeronasal 2, receptor 26(Vmn2r26)	1.8
AK007819		1.8
AK010512	DEAH (Asp-Glu-Ala-His) box polypeptide 40(Dhx40)	1.8
NM_010591	jun proto-oncogene(Jun)	1.8
D83144	sine oculis-related homeobox 3(Six3)	1.8
AK015711	RIKEN cDNA 4930505O20 gene(4930505O20Rik)	1.8
NM_026106	down-regulator of transcription 1(Dr1)	1.8
BC006674	spindle apparatus coiled-coil protein 1(Spdl1)	1.8

L24755	bone morphogenetic protein 1(Bmp1)	1.8
AJ298841	torsin family 1, member A (torsin A)(Tor1a)	1.8
NM_011604	toll-like receptor 6(Tlr6)	1.8
AK017233	RIKEN cDNA 5330403D14 gene(5330403D14Rik)	1.8
U84407	agrin(Agrn)	1.8
NM_009192	src-like adaptor(Sla)	1.8
AK007756	NADH dehydrogenase (ubiquinone) 1 alpha subcomplex assembly factor 8(Ndufaf8)	1.8
AK017945	RIKEN cDNA 5830424K16 gene(5830424K16Rik)	1.8
AK009622	RIKEN cDNA 2310034O05 gene(2310034O05Rik)	1.8
BC006061	RIKEN cDNA 2010111I01 gene(2010111I01Rik)	1.8
M12308		1.8
NM_025628	cytochrome c oxidase, subunit VIb polypeptide 1(Cox6b1)	1.8
D87034	teneurin transmembrane protein 4(Tenm4)	1.8
AK006691	RIKEN cDNA 1700042O13 gene(1700042O13Rik)	1.8
AY028960		1.8
NM_009975	casein kinase 2, beta polypeptide(Csnk2b)	1.8
AK007055	RIKEN cDNA 1700093P08 gene(1700093P08Rik)	1.8
AK016392	RIKEN cDNA 4930594M17 gene(4930594M17Rik)	1.8
AK015500	RIKEN cDNA 4930463O16 gene(4930463O16Rik)	1.8
AF053473	kinesin family member 5A(Kif5a)	1.8
AK019887	RIKEN cDNA 5033405D04 gene(5033405D04Rik)	1.8
AK015559		1.8
AF312938	activating transcription factor 5(Atf5)	1.8
AF366393	protein phosphatase 2, regulatory subunit B, delta(Ppp2r2d)	1.8
NM_026369	actin related protein 2/3 complex, subunit 5(Arpc5)	1.8
AK009628	RIKEN cDNA 2310035C23 gene(2310035C23Rik)	1.8
X16955		1.8
NM_025714	outer dense fiber of sperm tails 2-like(Odf2l)	1.8

AK016640	tousled-like kinase 2 (Arabidopsis)(Tlk2)	1.8
AF408408	sorting nexin 18(Snx18)	1.8
AK008865		1.8
AK021277	RIKEN cDNA C630043D15 gene(C630043D15Rik)	1.8
BC010479	crystallin, zeta (quinone reductase)-like 1(Cryzl1)	1.8
NM_008719	neuronal PAS domain protein 2(Npas2)	1.8
NM_016872	vesicle-associated membrane protein 5(Vamp5)	1.8
AK011290	phosphoribosyl pyrophosphate synthetase-associated protein 1(Prpsap1)	1.8
AY046504	translocated promoter region, nuclear basket protein(Tpr)	1.8
AF012178		1.8
U50718	nitric oxide synthase 1, neuronal(Nos1)	1.8
NM_011839	mab-21-like 2 (C. elegans)(Mab21l2)	1.8
AK018659	RIKEN cDNA 9130403I23 gene(9130403I23Rik)	1.9
AK013132	oxidative-stress responsive 1(Oxsr1)	1.9
AK016830	zinc finger protein 689(Zfp689)	1.9
AK016044	HYDIN, axonemal central pair apparatus protein(Hydin)	1.9
X17683		1.9
Z12214		1.9
AK014187	RIKEN cDNA 5430402O13 gene(5430402O13Rik)	1.9
AF384559		1.9
M15442	proteolipid protein (myelin) 1(Plp1)	1.9
AK012748	hydroxyacylglutathione hydrolase-like(Haghl)	1.9
NM_019999	paroxysmal nonkinesiogenic dyskinesia(Pnkd)	1.9
AK011950	RIKEN cDNA 2610301B20 gene(2610301B20Rik)	1.9
AK020352	serine peptidase inhibitor, Kazal type 12(Spink12)	1.9
AK019628	RIKEN cDNA 4930452N14 gene(4930452N14Rik)	1.9
AK011332	histone deacetylase 8(Hdac8)	1.9
NM_015772	sal-like 2 (Drosophila)(Sall2)	1.9

BC012262	Rho guanine nucleotide exchange factor (GEF) 3(Arhgef3)	1.9
AK012773	acyl-Coenzyme A dehydrogenase family, member 9(Acad9)	1.9
BC014857	serine/arginine-rich splicing factor 7(Srsf7)	1.9
AF131137		1.9
NM_024258	ubiquitin specific peptidase 16(Usp16)	1.9
AK020039	zinc finger with KRAB and SCAN domains 1(Zkscan1)	1.9
AK020189	zinc finger protein 626(Zfp626)	1.9
AF177147		1.9
BC004839	casein kinase 1, gamma 2(Csnk1g2)	1.9
AK018401	alpha-kinase 1(Alpk1)	1.9
NM_021565	midnolin(Midn)	1.9
AK013171	RIKEN cDNA 2810427C15 gene(2810427C15Rik)	1.9
AK018211	RIKEN cDNA 6330510M09 gene(6330510M09Rik)	1.9
AK017574	RIKEN cDNA 5730419A17 gene(5730419A17Rik)	1.9
NM_027028	tescalcin-like(Tescl)	1.9
AK004227	double homeobox B-like 1(Duxbl1)	1.9
Z12237		1.9
AK008693	zinc finger protein 846(Zfp846)	1.9
NM_019499	MAD2 mitotic arrest deficient-like 1(Mad2l1)	1.9
NM_010059	DNA meiotic recombinase 1(Dmc1)	1.9
AK014892	5'-nucleotidase, cytosolic IB(Nt5c1b)	1.9
BC008229	arginine/serine-rich coiled-coil 2(Rsrc2)	1.9
AK013012	kelch-like 35(Klhl35)	1.9
AK009282	LY6/PLAUR domain containing 6B(Lypd6b)	1.9
AK017765	RIKEN cDNA 5730512F23 gene(5730512F23Rik)	1.9
AK010706	ELAV (embryonic lethal, abnormal vision)-like 1 (Hu antigen R)(Elavl1)	1.9
BC011475	seizure related 6 homolog like 2(Sez6l2)	1.9
AK013767	RIKEN cDNA 2900072G19 gene(2900072G19Rik)	1.9

Z47780		1.9
U47923	platelet derived growth factor receptor, alpha polypeptide(Pdgfra)	1.9
NM_018852	sodium channel, voltage-gated, type IX, alpha(Scn9a)	1.9
AK009507	phospholipase A2, group IVE(Pla2g4e)	1.9
AK006234	centromere protein P(Cenpp)	1.9
NM_025780	THAP domain containing, apoptosis associated protein 2(Thap2)	1.9
AK004814	glutaminyl-tRNA synthetase(Qars)	1.9
AK015050	RIKEN cDNA 4930402H24 gene(4930402H24Rik)	1.9
NM_011716	Wolfram syndrome 1 homolog (human)(Wfs1)	1.9
NM_020612		1.9
AK004733	DEAH (Asp-Glu-Ala-His) box polypeptide 34(Dhx34)	1.9
AK009930	EPS8-like 1(Eps8l1)	1.9
AF145957	transducin-like enhancer of split 6(Tle6)	1.9
AF236125	immunoglobulin superfamily, DCC subclass, member 3(Igdcc3)	1.9
BC003901	cyclin-dependent kinase 9 (CDC2-related kinase)(Cdk9)	1.9
AF363457	caspase recruitment domain family, member 14(Card14)	1.9
BC003461	protein kinase, cAMP dependent regulatory, type I, alpha(Prkar1a)	1.9
X01433		1.9
M29246		1.9
AK014130	RWD domain containing 3(Rwdd3)	1.9
NM_029239	protein kinase D3(Prkd3)	1.9
AK013201	hematological and neurological expressed 1-like(Hn1l)	1.9
NM_028842	ring finger protein 138, retrogene 1(Rnf138rt1)	1.9
AK020460	RIKEN cDNA 9430038I01 gene(9430038I01Rik)	1.9
AK014123	RIKEN cDNA 3110035E14 gene(3110035E14Rik)	1.9
NM_021462	MAP kinase-interacting serine/threonine kinase 2(Mknk2)	1.9
AK011993	SET domain containing 3(Setd3)	1.9
AK015273	sedoheptulokinase(Shpk)	1.9

L14353		1.9
AK017623	RIKEN cDNA 5730437C11 gene(5730437C11Rik)	1.9
BC013508	mitotic spindle positioning(Misp)	1.9
AK010393	GPN-loop GTPase 1(Gpn1)	1.9
U50962	CTD (carboxy-terminal domain, RNA polymerase II, polypeptide A) small phosphatase 1(Ctdsp1)	1.9
AK007857	4short chain dehydrogenase/reductase family 9C, member 7(Sdr9c7)	1.9
BC004049	small integral membrane protein 15(Smim15)	1.9
AK007749	vacuolar protein sorting 13B(Vps13b)	1.9
AK004187	kelch-like 24(Klhl24)	1.9
AK007571	RIKEN cDNA 1810021B22 gene(1810021B22Rik)	1.9
NM_009084	ribosomal protein L37a(Rpl37a)	1.9
NM_009933	collagen, type VI, alpha 1(Col6a1)	1.9
AK017236	RIKEN cDNA 5330406M23 gene(5330406M23Rik)	1.9
AK019863	small nuclear RNA activating complex, polypeptide 3(Snapc3)	1.9
AK004390	sema domain, transmembrane domain (TM), and cytoplasmic domain, (semaphorin) 6D(Sema6d)	1.9
NM_009893	chordin(Chrd)	1.9
AF153350	a disintegrin and metallopeptidase domain 28(Adam28)	1.9
AK020508	RIKEN cDNA 9430087B13 gene(9430087B13Rik)	1.9
Z12390		1.9
U00941		1.9
AK016940	FYVE, RhoGEF and PH domain containing 6(Fgd6)	1.9
AK018257	RAB39B, member RAS oncogene family(Rab39b)	1.9
AB049941	mitochondrial ribosomal protein S5(Mrps5)	1.9
M17472		1.9
AK013984	ABRA C-terminal like(Abracl)	1.9
NM_026142	RIKEN cDNA 3632451O06 gene(3632451O06Rik)	1.9
D16599		1.9
X73014		1.9

BC012519	tubulin, gamma complex associated protein 2(Tubgcp2)	1.9
AK015953	RIKEN cDNA 4930533D04 gene(4930533D04Rik)	1.9
L19609		1.9
AK007102	RIKEN cDNA 1700100L14 gene(1700100L14Rik)	1.9
NM_031392	WD repeat domain 6(Wdr6)	1.9
AK009848	RIKEN cDNA 2310046G18 gene(2310046G18Rik)	1.9
NM_026306	tRNA methyltransferase 11-2(Trmt112)	1.9
AK017435	heart development protein with EGF-like domains 1(Heg1)	1.9
Z12273		1.9
AK015118	RIKEN cDNA 4930412E21 gene(4930412E21Rik)	1.9
AK014475	bromodomain containing 8(Brd8)	1.9
AK009865	DAZ interacting protein 3, zinc finger(Dzip3)	1.9
BC011162	neurocalcin delta(Ncald)	1.9
NM_030555	pre B cell leukemia homeobox 4(Pbx4)	1.9
AK010419	leucine rich repeat protein 1(Lrr1)	1.9
BC006828	microfibrillar-associated protein 3(Mfap3)	1.9
NM_007611	caspase 7(Casp7)	1.9
Z86015		1.9
AK012965	RIKEN cDNA 2810402E24 gene(2810402E24Rik)	1.9
AK008716	prostate androgen-regulated mucin-like protein 1(Parm1)	1.9
AK020061	RIKEN cDNA 6030442E23 gene(6030442E23Rik)	1.9
NM_022883	lipin 3(Lpin3)	1.9
AK016948	RIKEN cDNA 4933427E13 gene(4933427E13Rik)	1.9
NM_011157	serglycin(Srgn)	1.9
NM_013700	ubiquitin specific peptidase 5 (isopeptidase T)(Usp5)	1.9
AB009392	heterogeneous nuclear ribonucleoprotein L(Hnrnpl)	2.0
AK015598	RIKEN cDNA 4930480E11 gene(4930480E11Rik)	2.0
AK010472	zinc finger SWIM-type containing 7(Zswim7)	2.0

AK003069	RIKEN cDNA 0910001E24 gene(0910001E24Rik)	2.0
AK020702	PYM homolog 1, exon junction complex associated factor(Pym1)	2.0
AK015924	meiosis specific with OB domains(Meiob)	2.0
NM_008745	neurotrophic tyrosine kinase, receptor, type 2(Ntrk2)	2.0
U43520	dystrophin related protein 2(Drp2)	2.0
AK010219	autophagy/beclin 1 regulator 1(Ambra1)	2.0
AF102520	olfactory receptor 46(Olfr46)	2.0
NM_010507	interferon alpha 9(Ifna9)	2.0
AK011279	aurora kinase A and ninein interacting protein(Aunip)	2.0
AK010002	coiled-coil domain containing 115(Ccdc115)	2.0
NM_025602	coiled-coil domain containing 59(Ccdc59)	2.0
AK010574	RIKEN cDNA 2410022M11 gene(2410022M11Rik)	2.0
NM_020491	Sjogren's syndrome/scleroderma autoantigen 1 homolog (human)(Sssca1)	2.0
NM_027218	C-type lectin domain family 4, member b1(Clec4b1)	2.0
AF031634		2.0
AK010328	NADH dehydrogenase (ubiquinone) 1 alpha subcomplex, assembly factor 1(Ndufaf1)	2.0
AK015179	zinc finger protein 949(Zfp949)	2.0
AK011819	RIKEN cDNA 2610104F20 gene(2610104F20Rik)	2.0
AF041926		2.0
AF156549	ATPase, class V, type 10A(Atp10a)	2.0
NM_016697	glypican 3(Gpc3)	2.0
AK018518	family with sequence similarity 217, member B(Fam217b)	2.0
AF093874		2.0
NM_008088	growth arrest specific 7(Gas7)	2.0
AK006762	cut-like homeobox 2(Cux2)	2.0
S42582		2.0
D29936		2.0
AK015477	RIKEN cDNA 4930456J16 gene(4930456J16Rik)	2.0

AK018831	RIKEN cDNA 4930471G03 gene(4930471G03Rik)	2.0
NM_011351	sema domain, transmembrane domain (TM), and cytoplasmic domain, (semaphorin) 6C(Sema6c)	2.0
M22733		2.0
NM_010656	sarcospan(Sspn)	2.0
AK003240	sodium channel, voltage-gated, type III, beta(Scn3b)	2.0
AK009101	COMM domain containing 10(Commd10)	2.0
AK019708	RIKEN cDNA 4930528J18 gene(4930528J18Rik)	2.0
AK015120	RIKEN cDNA 4930412F12 gene(4930412F12Rik)	2.0
NM_009980	C-terminal binding protein 2(Ctbp2)	2.0
AK020720	TATA-box binding protein associated factor, RNA polymerase I, B(Taf1b)	2.0
AF000911		2.0
BC011111	signal sequence receptor, gamma(Ssr3)	2.0
M27134		2.0
AK011204	TBC1 domain family, member 13(Tbc1d13)	2.0
AK016318	RIKEN cDNA 4930579F01 gene(4930579F01Rik)	2.0
AF013578		2.0
AJ006521	cholinergic receptor, muscarinic 1, CNS(Chrm1)	2.0
AK016759	centrosomal protein 350(Cep350)	2.0
NM_010280	glial cell line derived neurotrophic factor family receptor alpha 3(Gfra3)	2.0
AK007061	dual specificity phosphatase 21(Dusp21)	2.0
NM_009794	calpain 2(Capn2)	2.0
AK006168	E1A binding protein p400(Ep400)	2.0
AK016667	GINS complex subunit 4 (Sld5 homolog)(Gins4)	2.0
BC003209	integrator complex subunit 3(Ints3)	2.0
AK019902	RIKEN cDNA 5330421F21 gene(5330421F21Rik)	2.0
AK007704	expressed sequence AU040320(AU040320)	2.0
AK018327	G protein-coupled receptor 165(Gpr165)	2.0
AK012513	RIKEN cDNA C630043F03 gene(C630043F03Rik)	2.0

X73024		2.0
AK012128	MPN domain containing(Mpnd)	2.0
AF245444	glutamate receptor, ionotropic, kainate 3(Grik3)	2.0
AK016576		2.0
AK015872	sorting nexin 16(Snx16)	2.0
AF326297		2.0
Z12544		2.0
AK012725	sperm antigen with calponin homology and coiled-coil domains 1(Specc1)	2.0
AK002276	all-trans retinoic acid induced differentiation factor(Atraid)	2.0
M64157		2.0
M32072	retinoic acid receptor, gamma(Rarg)	2.0
AK018881	RIKEN cDNA 1700066C05 gene(1700066C05Rik)	2.0
NM_008334	interferon alpha 7(Ifna7)	2.0
AK005480	RIKEN cDNA 1600017G08 gene(1600017G08Rik)	2.0
AK005642	RIKEN cDNA 1700003I16 gene(1700003I16Rik)	2.0
AK021098	gamma-aminobutyric acid (GABA) A receptor, subunit beta 2(Gabrb2)	2.0
BC004035	ADP-ribosylation factor-like 8A(Arl8a)	2.0
AK011544	DIM1 dimethyladenosine transferase 1-like (S. cerevisiae)(Dimt1)	2.0
NM_010865	myocilin(Myoc)	2.0
AK016638	twist basic helix-loop-helix transcription factor 1 neighbor(Twistnb)	2.0
NM_019416		2.0
X70182	enolase 3, beta muscle(Eno3)	2.0
Z12431		2.0
AK012792	RIKEN cDNA 1700037C18 gene(1700037C18Rik)	2.0
AK004339	SR-related CTD-associated factor 11(Scaf11)	2.0
NM_017372	lysozyme 2(Lyz2)	2.0
AK009671	carboxylesterase 2F(Ces2f)	2.0
AF041889		2.0

NM_010800	basic helix-loop-helix family, member a15(Bhlha15)	2.0
AK011687	RIKEN cDNA 2610036A22 gene(2610036A22Rik)	2.0
NM_013886	hepatoma-derived growth factor, related protein 3(Hdgfrp3)	2.0
AK015694	RIKEN cDNA 4930504B16 gene(4930504B16Rik)	2.0
AK006139	RIKEN cDNA 1700019P21 gene(1700019P21Rik)	2.0
AF148638	pelota homolog (Drosophila)(Pelo)	2.0
BC009104	transcription elongation factor B (SIII), polypeptide 1(Tceb1)	2.1
U26784		2.1
AK004470	urate (5-hydroxyiso-) hydrolase(Urah)	2.1
AF357489		2.1
BC005716	acylpeptide hydrolase(Apeh)	2.1
NM_031179	splicing factor 3b, subunit 1(Sf3b1)	2.1
AK020935	RIKEN cDNA A930036K24 gene(A930036K24Rik)	2.1
AF075717	TGF-beta1-induced anti-apoptotic factor 2(Tiaf2)	2.1
NM_021386	claudin 10(Cldn10)	2.1
AY046077	Notch-regulated ankyrin repeat protein(Nrarp)	2.1
BC014805	solute carrier family 22 (organic cation transporter), member 26(Slc22a26)	2.1
AJ400977		2.1
AK006701	RIKEN cDNA 1700046C09 gene(1700046C09Rik)	2.1
AF093135	interleukin-1 receptor-associated kinase 1 binding protein 1(Irak1bp1)	2.1
NM_020620		2.1
U21477		2.1
BC010539	aarF domain containing kinase 1(Adck1)	2.1
AK015123	RIKEN cDNA 4930412O06 gene(4930412O06Rik)	2.1
AK011987	fem-1 homolog c (C.elegans)(Fem1c)	2.1
AF095938	succinate dehydrogenase complex, subunit A, flavoprotein (Fp)(Sdha)	2.1
BC011531		2.1
U35650	solute carrier family 7 (cationic amino acid transporter, y+ system), member 2(Slc7a2)	2.1

NM_007910	ephrin A4(Efna4)	2.1
BC004710	target of myb1-like 1 (chicken)(Tom1l1)	2.1
AB047921	GATA zinc finger domain containing 1(Gatad1)	2.1
AK012547	structural maintenance of chromosomes 2, opposite strand(Smc2os)	2.1
NM_025577	RIKEN cDNA 2810428I15 gene(2810428I15Rik)	2.1
M24272		2.1
NM_008137	guanine nucleotide binding protein, alpha 14(Gna14)	2.1
AK015244	RIKEN cDNA 4930429N05 gene(4930429N05Rik)	2.1
NM_019823	cytochrome P450, family 2, subfamily d, polypeptide 22(Cyp2d22)	2.1
AK008691	RIKEN cDNA 2210009P08 gene(2210009P08Rik)	2.1
NM_010128	epithelial membrane protein 1(Emp1)	2.1
AK020947	RIKEN cDNA A930040O22 gene(A930040O22Rik)	2.1
M37595		2.1
U26798		2.1
AK018071	leucine rich repeat containing 17(Lrrc17)	2.1
NM_008335		2.1
AK007177	RIKEN cDNA 1700112H15 gene(1700112H15Rik)	2.1
NM_009591	arylalkylamine N-acetyltransferase(Aanat)	2.1
AK007425	RIKEN cDNA 1810010K12 gene(1810010K12Rik)	2.1
AF318455		2.1
AK017739	protein kinase D3(Prkd3)	2.1
AA204426		2.1
NM_007925	elastin(Eln)	2.1
NM_013684	TATA box binding protein(Tbp)	2.1
AK008602	von Willebrand factor A domain containing 9(Vwa9)	2.1
AK019723	RIKEN cDNA 4930538L07 gene(4930538L07Rik)	2.1
AJ231232		2.1
NM_009370	transforming growth factor, beta receptor I(Tgfbr1)	2.1

AK005888	RIKEN cDNA 1700012B07 gene(1700012B07Rik)	2.1
AK021081	RIKEN cDNA C030014I23 gene(C030014I23Rik)	2.1
NM_019518	GRP1 (general receptor for phosphoinositides 1)-associated scaffold protein(Grasp)	2.1
AF220294		2.1
AK014665	peptidyl prolyl isomerase H(Ppih)	2.1
NM_025368	Josephin domain containing 2(Josd2)	2.1
AK005699	solute carrier family 16 (monocarboxylic acid transporters), member 13(Slc16a13)	2.1
NM_026527	ChaC, cation transport regulator 2(Chac2)	2.1
AK007785	zinc finger protein 335(Zfp335)	2.1
NM_021525	RNA terminal phosphate cyclase-like 1(Rcl1)	2.1
AK007503	YdjC homolog (bacterial)(Ydjc)	2.1
NM_011019	oncostatin M receptor(Osmr)	2.1
AK015547	RIKEN cDNA 5430427N15 gene(5430427N15Rik)	2.1
NM_010787	male enhanced antigen 1(Mea1)	2.1
AK014235	exportin, tRNA (nuclear export receptor for tRNAs)(Xpot)	2.1
AK020581	solute carrier family 30 (zinc transporter), member 6(Slc30a6)	2.1
BC011457	transmembrane and coiled-coil domains 1(Tmco1)	2.1
X02937		2.2
AK021029	DNA segment, Chr 4, ERATO Doi 617, expressed(D4Ertd617e)	2.2
AK017346	forkhead box N3(Foxn3)	2.2
AK014672	GLIS family zinc finger 3(Glis3)	2.2
NM_015814	dickkopf WNT signaling pathway inhibitor 3(Dkk3)	2.2
AK007343	heat shock factor binding protein 1-like 1(Hsbp1l1)	2.2
AK008688	cytochrome P450, family 2, subfamily c, polypeptide 65(Cyp2c65)	2.2
AB072395	topoisomerase I binding, arginine/serine-rich(Topors)	2.2
NM_009033	RNA binding motif protein, X-linked like-1(Rbmxl1)	2.2
NM_025407	ubiquinol-cytochrome c reductase core protein 1(Uqcrc1)	2.2
NM_008836	per-hexamer repeat gene 5(Phxr5)	2.2

AK014874	CDKN2A interacting protein(Cdkn2aip)	2.2
AK017337	solute carrier family 6 (neurotransmitter transporter), member 19, opposite strand(Slc6a19os)	2.2
U20263	coatomer protein complex, subunit gamma 2, opposite strand 2(Copg2os2)	2.2
AK007700	syntaxin 18(Stx18)	2.2
AK006889	RIKEN cDNA 1700065D16 gene(1700065D16Rik)	2.2
AJ293897	ribonuclease P/MRP 25 subunit-like(Rpp25l)	2.2
AF110764	dCTP pyrophosphatase 1(Dctpp1)	2.2
AK006890	spermatogenesis associated 17(Spata17)	2.2
X99722	histone cell cycle regulator(Hira)	2.2
AK010736	zinc finger and BTB domain containing 8a(Zbtb8a)	2.2
AK016876	RIKEN cDNA 4933423L19 gene(4933423L19Rik)	2.2
BC009090	tumor necrosis factor, alpha-induced protein 8(Tnfaip8)	2.2
NM_019702	Hbs1-like (S. cerevisiae)(Hbs1l)	2.2
AF145962		2.2
AK015232	RIKEN cDNA 4930429C20 gene(4930429C20Rik)	2.2
AF050429		2.2
AK019682	RIKEN cDNA 4930517L18 gene(4930517L18Rik)	2.2
AK003420	cell growth regulator with EF hand domain 1(Cgref1)	2.2
AK020872	DNA segment, Chr 5, ERATO Doi 579, expressed(D5Ertd579e)	2.2
AK011896	ring finger protein 219(Rnf219)	2.2
AK003791	olfactomedin-like 2B(Olfml2b)	2.2
AK007013	RIKEN cDNA 1700086D15 gene(1700086D15Rik)	2.2
AK019620	RIKEN cDNA 4930449E18 gene(4930449E18Rik)	2.2
NM_009582	mitogen-activated protein kinase kinase kinase 12(Map3k12)	2.2
AK016624	hexamethylene bis-acetamide inducible 2(Hexim2)	2.2
AK017191	RIKEN cDNA 5033425B01 gene(5033425B01Rik)	2.2
NM_008008	fibroblast growth factor 7(Fgf7)	2.2
BC006782	pre-mRNA processing factor 3(Prpf3)	2.2

NM_021447	tripartite motif-containing 54(Trim54)	2.2
BC005471	zinc finger protein 810(Zfp810)	2.2
NM_007419	adrenergic receptor, beta 1(Adrb1)	2.2
AK007154	RIKEN cDNA 1700109K24 gene(1700109K24Rik)	2.2
AK006942	RIKEN cDNA 1700072O05 gene(1700072O05Rik)	2.2
AK007219	RIKEN cDNA 1700120O09 gene(1700120O09Rik)	2.2
NM_020517	lens epithelial protein(Lenep)	2.2
AK004481	valyl-tRNA synthetase 2, mitochondrial(Vars2)	2.2
AK018075	prickle planar cell polarity protein 2(Prickle2)	2.2
AK015766	ubiquitin specific peptidase 50(Usp50)	2.2
AK006729	RIKEN cDNA 1700048J15 gene(1700048J15Rik)	2.2
AK018532	RIKEN cDNA 9030601B04 gene(9030601B04Rik)	2.2
AK020407	RIKEN cDNA 9430007M09 gene(9430007M09Rik)	2.2
AY038079	F-box and WD-40 domain protein 11(Fbxw11)	2.2
AK019805	RIKEN cDNA 4930571O06 gene(4930571O06Rik)	2.2
NM_016681	checkpoint kinase 2(Chek2)	2.2
NM_008421	potassium voltage gated channel, Shaw-related subfamily, member 1(Kcnc1)	2.2
NM_025618	sorcin(Sri)	2.2
NM_025637	RWD domain containing 3(Rwdd3)	2.2
NM_010356	glutathione S-transferase, alpha 3(Gsta3)	2.2
BC010793	polymerase (RNA) III (DNA directed) polypeptide H(Polr3h)	2.2
BC009020	zinc finger, CCHC domain containing 14(Zcchc14)	2.2
NM_021272	fatty acid binding protein 7, brain(Fabp7)	2.2
NM_018856	cyclin L2(Ccnl2)	2.2
AK006533	RWD domain containing 2A(Rwdd2a)	2.2
AK018192	solute carrier family 24 (sodium/potassium/calcium exchanger), member 2(Slc24a2)	2.2
AK008694	transcription elongation factor A (SII) N-terminal and central domain containing 2(Tceanc2)	2.2
BC003867	formin binding protein 1(Fnbp1)	2.2

AJ409466		2.2
Z12229		2.2
NM_018743	glycerol-3-phosphate acyltransferase 4(Gpat4)	2.3
AK016148	RIKEN cDNA 4930556J24 gene(4930556J24Rik)	2.3
AK003833		2.3
NM_013465	alpha-2-HS-glycoprotein(Ahsg)	2.3
AK003578	SHANK-associated RH domain interacting protein(Sharpin)	2.3
AK014481	AT rich interactive domain 2 (ARID, RFX-like)(Arid2)	2.3
NM_025436	methylsterol monoxygenase 1(Msmo1)	2.3
AK017553	collagen, type XIV, alpha 1(Col14a1)	2.3
AK014924	RIKEN cDNA 4921519G19 gene(4921519G19Rik)	2.3
L15321		2.3
NM_009133	stathmin-like 3(Stmn3)	2.3
AK020111	RIKEN cDNA 6720418B01 gene(6720418B01Rik)	2.3
NM_013617	olfactory receptor 64(Olfr64)	2.3
BC013544	thioredoxin-related transmembrane protein 2(Tmx2)	2.3
AK014851	family with sequence similarity 71, member D(Fam71d)	2.3
NM_007617	caveolin 3(Cav3)	2.3
AK014068	solute carrier family 25 (mitochondrial carrier, Graves disease autoantigen), member 16(Slc25a16)	2.3
AK018382	zinc finger, ZZ-type with EF hand domain 1(Zzef1)	2.3
AK020279	RIKEN cDNA 9130214F15 gene(9130214F15Rik)	2.3
NM_019794	DnaJ heat shock protein family (Hsp40) member A2(Dnaja2)	2.3
X86534		2.3
AK015366	RIKEN cDNA 4930442P07 gene(4930442P07Rik)	2.3
AK002772	Purkinje cell protein 4-like 1(Pcp4l1)	2.3
U55685		2.3
AF033565	CDC-like kinase 3(Clk3)	2.3
AK016769	RIKEN cDNA 4933411E08 gene(4933411E08Rik)	2.3

AF357423	small nucleolar RNA, H/ACA box 35(Snora35)	2.3
U13262	myelin basic protein expression factor 2, repressor(Myef2)	2.3
NM_025437	eukaryotic translation initiation factor 1A, X-linked(Eif1ax)	2.3
AK015268	RIKEN cDNA 4930431H11 gene(4930431H11Rik)	2.3
BC003286	ankyrin repeat domain 13a(Ankrd13a)	2.3
AK012913	B cell CLL/lymphoma 11A (zinc finger protein)(Bcl11a)	2.3
NM_009154	sema domain, seven thrombospondin repeats (type 1 and type 1-like), transmembrane domain (TM) and short cytoplasmic domain, (semaphorin) 5A(Sema5a)	2.3
NM_009046	avian reticuloendotheliosis viral (v-rel) oncogene related B(Relb)	2.3
NM_024189	YY1 associated factor 2(Yaf2)	2.3
AK021129	RIKEN cDNA 9530077C05 gene(9530077C05Rik)	2.3
AK018179		2.3
NM_016701	nestin(Nes)	2.3
AK015348	ubiquitin-conjugating enzyme E2W (putative)(Ube2w)	2.3
Z12271		2.3
NM_023788	melanoma antigen, family H, 1(Mageh1)	2.3
AK011000	peptidylprolyl isomerase (cyclophilin)-like 3(Ppil3)	2.3
X73020		2.3
AK006052	RIKEN cDNA 1700016P04 gene(1700016P04Rik)	2.3
AB041610	component of oligomeric golgi complex 8(Cog8)	2.3
AK015230		2.3
AY033886	solute carrier family 5 (sodium/glucose cotransporter), member 2(Slc5a2)	2.3
AK007529	RIKEN cDNA 1810017P11 gene(1810017P11Rik)	2.3
NM_013493	cellular nucleic acid binding protein(Cnbp)	2.3
AK015846	RIKEN cDNA 4930519N06 gene(4930519N06Rik)	2.3
AK008096	cytochrome c oxidase assembly protein 11(Cox11)	2.3
BC004614	mitochondrial ribosomal protein L30(Mrpl30)	2.4
BC003941	zinc finger protein 639(Zfp639)	2.4

AF357317	small nucleolar RNA, C/D box 17(Snord17)	2.4
AF331708	natriuretic peptide receptor 2(Npr2)	2.4
AK020996	RIKEN cDNA B230207M22 gene(B230207M22Rik)	2.4
D29770		2.4
U29496	zinc finger protein 82(Zfp82)	2.4
NM_010676	keratin associated protein 19-5(Krtap19-5)	2.4
NM_026021	zinc finger, MYND domain containing 19(Zmynd19)	2.4
AB041263	N-acetylneuraminic acid synthase (sialic acid synthase)(Nans)	2.4
AK014137	RIKEN cDNA 3110038A09 gene(3110038A09Rik)	2.4
NM_016682	ubiquitin-like modifier activating enzyme 2(Uba2)	2.4
NM_026925	pancreatic lipase(Pnlip)	2.4
NM_016843	ataxin 10(Atxn10)	2.4
AK003497	SRY (sex determining region Y)-box 6, opposite strand(Sox6os)	2.4
AK007608	RIKEN cDNA 1810026J23 gene(1810026J23Rik)	2.4
X14930		2.4
AK020016	RIKEN cDNA 5830420C07 gene(5830420C07Rik)	2.4
AK012831	DnaJ heat shock protein family (Hsp40) member C6(Dnajc6)	2.4
AJ011107	chloride channel, voltage-sensitive 1(Clcn1)	2.4
AK004069	TAO kinase 2(Taok2)	2.4
AB042155	zinc finger protein of the cerebellum 5(Zic5)	2.4
AK020460		2.4
AK009897	ataxia, cerebellar, Cayman type, opposite strand(Atcayos)	2.4
U48241	D4, zinc and double PHD fingers family 1(Dpf1)	2.4
AK019113	lipase, member H(Liph)	2.4
AK007270	sperm acrosome associated 1(Spaca1)	2.4
BC011383	HECT and RLD domain containing E3 ubiquitin protein ligase family member 1(Herc1)	2.4
NM_026804	cell death-inducing DNA fragmentation factor, alpha subunit-like effector B(Cideb)	2.4
L37148		2.4

AK020216	RIKEN cDNA 8030479D07 gene(8030479D07Rik)	2.4
AF039839		2.4
D12728		2.4
Z12521		2.4
AF220209	Nedd4 family interacting protein 1(Ndfip1)	2.4
U05819		2.4
NM_025895	mediator complex subunit 28(Med28)	2.4
AK004572	poly(A)-specific ribonuclease (deadenylation nuclease)(Parn)	2.4
NM_009491	vomeronasal 2, receptor 10(Vmn2r10)	2.4
AF149291	transgelin 2(Tagln2)	2.5
AK003083	syncollin(Sycn)	2.5
M34525		2.5
NM_008250	H2.0-like homeobox(Hlx)	2.5
AB041601	transmembrane protein 191C(Tmem191c)	2.5
AK012318		2.5
NM_009146	ferric-chelate reductase 1(Frrs1)	2.5
BC014738	methionine sulfoxide reductase A(Msra)	2.5
Y10969		2.5
NM_013566	integrin beta 7(Itgb7)	2.5
AK009638	polycomb group ring finger 3(Pcgf3)	2.5
AK014909	RIKEN cDNA 4921517D22 gene(4921517D22Rik)	2.5
L10895	prostate tumor over expressed gene 1(Ptov1)	2.5
AK010014	interferon, alpha-inducible protein 27 like 2A(Ifi27l2a)	2.5
AK009420	ring finger protein 113A2(Rnf113a2)	2.5
AF011413	vomeronasal 2, receptor 88(Vmn2r88)	2.5
AK007057	ubiquitin specific peptidase 46, opposite strand 1(Usp46os1)	2.5
AK018947	RIKEN cDNA 1700109O10 gene(1700109O10Rik)	2.5
AK020596	RIKEN cDNA 9530046B11 gene(9530046B11Rik)	2.5

AK015560	zinc finger protein 572(Zfp572)	2.5
NM_007640	CD1d2 antigen(Cd1d2)	2.5
NM_011274	URI1, prefoldin-like chaperone(Uri1)	2.5
AJ272163	estrogen receptor 1 (alpha)(Esr1)	2.6
S51858	calcium binding protein 39(Cab39)	2.6
AF398971	ankyrin repeat and SOCS box-containing 10(Asb10)	2.6
AK014912	cyclic nucleotide binding domain containing 2(Cnbd2)	2.6
BC009022	isocitrate dehydrogenase 3 (NAD+) beta(Idh3b)	2.6
X55507		2.6
AK010042	serine peptidase inhibitor, Kazal type 5(Spink5)	2.6
AK015055	RIKEN cDNA 4930402N20 gene(4930402N20Rik)	2.6
AK008951		2.6
U78502		2.6
U05672	adenosine A2a receptor(Adora2a)	2.6
AB035381	ATPase type 13A1(Atp13a1)	2.6
AK019990	homeobox A3(Hoxa3)	2.6
NM_024264	cytochrome P450, family 27, subfamily a, polypeptide 1(Cyp27a1)	2.6
AF012154		2.6
AK014062	RIKEN cDNA 3110018I06 gene(3110018I06Rik)	2.6
NM_008216	hyaluronan synthase 2(Has2)	2.7
AK008065	transmembrane protein 236(Tmem236)	2.7
AJ007734	small nucleolar RNA, C/D box 7(Snord7)	2.7
AK015042	RIKEN cDNA 4930402C01 gene(4930402C01Rik)	2.7
AK012934	germ cell-less, spermatogenesis associated 1(Gmcl1)	2.7
NM_008873	plasminogen activator, urokinase(Plau)	2.7
AF349144		2.7
AK009225	shisa family member 5(Shisa5)	2.7
NM_010727	ligand of numb-protein X 1(Lnx1)	2.7

NM_019671	neuroepithelial cell transforming gene 1(Net1)	2.7
NM_011826	HCLS1 associated X-1(Hax1)	2.7
AK010745	biogenesis of lysosomal organelles complex-1, subunit 2(Bloc1s2)	2.7
NM_009001	RAB3A, member RAS oncogene family(Rab3a)	2.7
AK005250	solute carrier family 25 (mitochondrial carrier), member 18(Slc25a18)	2.7
AK017787	ubiquitin-like modifier activating enzyme 5(Uba5)	2.7
AK015748	RIKEN cDNA 4930511C11 gene(4930511C11Rik)	2.8
AF332067	TELO2 interacting protein 1(Tti1)	2.8
AK006144	testis-specific serine kinase 4(Tssk4)	2.8
AK018511	myosin IIIA(Myo3a)	2.8
NM_009144	secreted frizzled-related protein 2(Sfrp2)	2.8
AK016382	RIKEN cDNA 4930590L20 gene(4930590L20Rik)	2.8
NM_019637	serine/threonine/tyrosine interaction protein(Styx)	2.8
NM_007671	cyclin-dependent kinase inhibitor 2C (p18, inhibits CDK4)(Cdkn2c)	2.8
BC010981	cDNA sequence BC010981(BC010981)	2.8
NM_008809	platelet derived growth factor receptor, beta polypeptide(Pdgfrb)	2.8
AK019159	neogenin(Neo1)	2.8
AK010814	RIKEN cDNA 2410152P15 gene(2410152P15Rik)	2.8
AK006641	RIKEN cDNA 1700039E22 gene(1700039E22Rik)	2.8
NM_010916	nescient helix loop helix 1(Nhlh1)	2.8
BC011300	Fgfr1op N-terminal like(Fopnl)	2.8
AK019831	RIKEN cDNA 4930588D02 gene(4930588D02Rik)	2.8
BC011201	protein O-linked mannose beta 1,2-N-acetylglucosaminyltransferase(Pomgnt1)	2.8
NM_007801	cathepsin H(Ctsh)	2.9
AF301017	chemokine (C-X-C motif) ligand 16(Cxcl16)	2.9
NM_010929	notch 4(Notch4)	2.9
AK005238	suppressor of cytokine signaling 6(Socs6)	2.9
AF384100	mitofusin 2(Mfn2)	2.9

NM_008638	methylenetetrahydrofolate dehydrogenase (NAD+ dependent), methenyltetrahydrofolate cyclohydrolase(Mthfd2)	2.9
AK016169	RIKEN cDNA 4930558C23 gene(4930558C23Rik)	2.9
BC006036	HMG box domain containing 4(Hmgxb4)	2.9
AK014088	RIKEN cDNA 3110027N22 gene(3110027N22Rik)	2.9
AK008024	solute carrier family 7 (cationic amino acid transporter, y+ system), member 15(Slc7a15)	2.9
NM_009540	zinc finger protein, autosomal, pseudogene(Zfa-ps)	2.9
AK008392	calcineurin-like EF hand protein 2(Chp2)	2.9
M94308		3.0
NM_018854	intraflagellar transport 20(Ift20)	3.0
U39066	mitogen-activated protein kinase kinase 6(Map2k6)	3.0
AB026046	membrane-spanning 4-domains, subfamily A, member 10(Ms4a10)	3.0
NM_020564	sulfotransferase family 5A, member 1(Sult5a1)	3.0
NM_008258	hematological and neurological expressed sequence 1(Hn1)	3.0
NM_016749	myosin binding protein H(Mybph)	3.1
NM_021877	hairless(Hr)	3.1
NM_019912	ubiquitin-conjugating enzyme E2D 2A(Ube2d2a)	3.1
NM_028744	phosphatidylinositol 4-kinase type 2 beta(Pi4k2b)	3.1
AY042199		3.1
M16357	major urinary protein 3(Mup3)	3.1
AK014010	uncharacterized LOC100049077(LOC100049077)	3.1
NM_011144	peroxisome proliferator activated receptor alpha(Ppara)	3.1
NM_026275	ubiquitin-conjugating enzyme E2R 2(Ube2r2)	3.1
AK008511	upregulated during skeletal muscle growth 5(Usmg5)	3.1
AK020715	patched 1(Ptch1)	3.1
AK014156	RIKEN cDNA 2310057M21 gene(2310057M21Rik)	3.1
AY029528	exportin 6(Xpo6)	3.1
AK006081	RIKEN cDNA 1700018C11 gene(1700018C11Rik)	3.2

AJ132433	proline rich 15(Prr15)	3.2
NM_025634	transmembrane epididymal family member 3(Teddm3)	3.2
NM_012065	phosphodiesterase 6G, cGMP-specific, rod, gamma(Pde6g)	3.2
AK011425	solute carrier family 25 (mitochondrial carrier, phosphate carrier), member 24(Slc25a24)	3.2
AK014513	RIKEN cDNA 4631405K08 gene(4631405K08Rik)	3.2
U85993	Bardet-Biedl syndrome 9 (human)(Bbs9)	3.2
AK010096	angiomotin-like 1(Amotl1)	3.3
NM_029203	reproductive homeobox 2A(Rhox2a)	3.3
AY073184		3.3
BC007157	transmembrane protein 94(Tmem94)	3.3
AK002477	plasma membrane proteolipid(Pllp)	3.3
NM_010014	disabled 1(Dab1)	3.3
AK014620	RIKEN cDNA 4633402D09 gene(4633402D09Rik)	3.4
X02932		3.4
M82903	Myb-related transcription factor, partner of profilin(Mypop)	3.5
AK007111	RIKEN cDNA 1700102F20 gene(1700102F20Rik)	3.5
AK005374	ubiquinol-cytochrome c reductase, complex III subunit VII(Uqcrq)	3.6
AK002612	spectrin repeat containing, nuclear envelope family member 4(Syne4)	3.6
BC006938	phosphatidylinositol glycan anchor biosynthesis, class C(Pigc)	3.7
AJ409468		3.7
AK019163	zinc finger protein 623(Zfp623)	3.8
NM_025304	leucine carboxyl methyltransferase 1(Lcmt1)	3.8
NM_009346	TEA domain family member 1(Tead1)	4.0
NM_008090	GATA binding protein 2(Gata2)	4.1
X76850	MAP kinase-activated protein kinase 2(Mapkapk2)	4.1
AF237887	regulator of calcineurin 2(Rcan2)	4.1
NM_032005	T-box 19(Tbx19)	4.2
Z12755		4.3

AK016850	golgi autoantigen, golgin subfamily a, 7B(Golga7b)	4.3
AF230400	tripartite motif-containing 23(Trim23)	5.1
NM_010813	max binding protein(Mnt)	5.1
